# Non-suicidal self-injury is linked to mind-body intuitions: an exploratory study

**DOI:** 10.1038/s41598-026-73035-3

**Published:** 2026-10-22

**Authors:** Iris Berent, Chelsea Boccagno, Jill M. Hooley

**Affiliations:** 1 Northeastern University, Boston, USA; 2 Massachusetts General Hospital, Boston, USA; 3 Harvard T.H. Chan School of Public Health, Boston, USA; 4 Harvard University, Cambridge, USA

**Keywords:** Non-suicidal self-injury, Intuitive dualism, Psychological pain, Consciousness, Theory of mind, Affective processing, Neuroscience, Psychology, Psychology

## Abstract

Non-suicidal self-injury (NSSI) presents a pressing mind-body conundrum: why does the infliction of physical pain alleviate psychological suffering? We suggest that NSSI might arise, in part, from a difference in intuitive Dualism. We hypothesize that the tacit mind-body divide typically guides the conscious differentiation of physical and psychological pain. If, in NSSI, the mind-body divide is attenuated, psychological pain would be harder to recognize and differentiate from physical pain. Here, we evaluate this prediction in an exploratory online study of mind-body intuitions among individuals with NSSI engagement (*N* = 47) and Controls (*N* = 52). Results show that people with more severe lifetime NSSI view the psyche as more physical—more likely to persist by duplicating one’s body and desist upon the body’s demise. Furthermore, among participants with more severe NSSI, those who manifested a weaker mind-body divide were less likely to endorse established NSSI functions as explanations for their self-injury. Our results cannot determine whether intuitive Dualism plays a causal role in NSSI or NSSI motivations. Nonetheless, in this exploratory online sample, the mind–body judgments of people with more severe lifetime NSSI suggested stronger Physicalist intuitions. Thus, individual differences in intuitive Dualism could shed light on NSSI, and potentially, psychopathology more broadly.

**Supplementary Information:**

The online version contains supplementary material available at https://doi.org/10.1038/s41598-026-73035-3.

## Introduction

Non-suicidal self-injury (NSSI) is the deliberate, self-infliction of damage to bodily tissue without suicidal intent, by acts such as cutting, burning, or hitting^1^. NSSI is worrisome because it injures the body and increases risk for future death by suicide^1^. It is a highly prevalent behavior, affecting one in five adolescents globally^2,3^.

NSSI further presents a mind-body conundrum. When asked to explain why they engage in NSSI, many people mention that NSSI improves affect^1^. These affective benefits are puzzling. Logic would suggest that physical pain ought to exacerbate suffering. Why, then, does the self-infliction of bodily pain alleviate psychological anguish?

Here, we outline a novel explanation for this conundrum. We suggest that NSSI might arise, in part, from a difference in intuitive Dualism—the tacit separation of mind and body^4^. Our exploratory study, reported next, is the first to link NSSI engagement to differences in intuitive Dualism. Beyond its contribution to existing theories of NSSI, this research opens the possibility that individual differences in intuitive Dualism could shed light on psychopathology.

### The NSSI puzzle

Let us consider the NSSI puzzle: why does the infliction of physical pain alleviate psychological suffering in NSSI?

NSSI, no doubt, can certainly offer multiple *extrinsic* benefits, arising not from pain itself but rather because pain is a means to some other end, such as self-punishment^5,6^ or peer-bonding^7,8^. Pain, however, could also be beneficial for *intrinsic* reasons. It is these intrinsic benefits that we find most puzzling. How could pain be inherently beneficial?

Hooley and Franklin^6^ attribute these benefits to the phenomenon of “pain offset relief”. When one noxious stimulus follows a more intense noxious stimulus, the perception of pain decreases (relative to continuous stimulation^9^. The effect of NSSI on psychological pain could arise from the same pain-offset response mechanism: the physical pain resulting from NSSI could offset psychological pain, reducing negative affect and increasing positive affect^6^—possibly because physical and psychological pain rely on common brain circuitry^10–12^.

In the benefits and barrier model^6^, then, psychological and physical pain are homologous, as they trade off in the pain offset phenomenon. It is precisely because of this tradeoff relation that physical pain, in this model, is beneficial. But in solving the NSSI conundrum, this model opens up another puzzle. If physical and psychological pain are interchangeably *linked* (as they both rely on a shared neural mechanism), then why do people conceive them as *distinct*? What cognitive mechanisms allow us to contrast psychological and physical pain? And what role might these mechanisms play in NSSI?

**Fig. 1:**
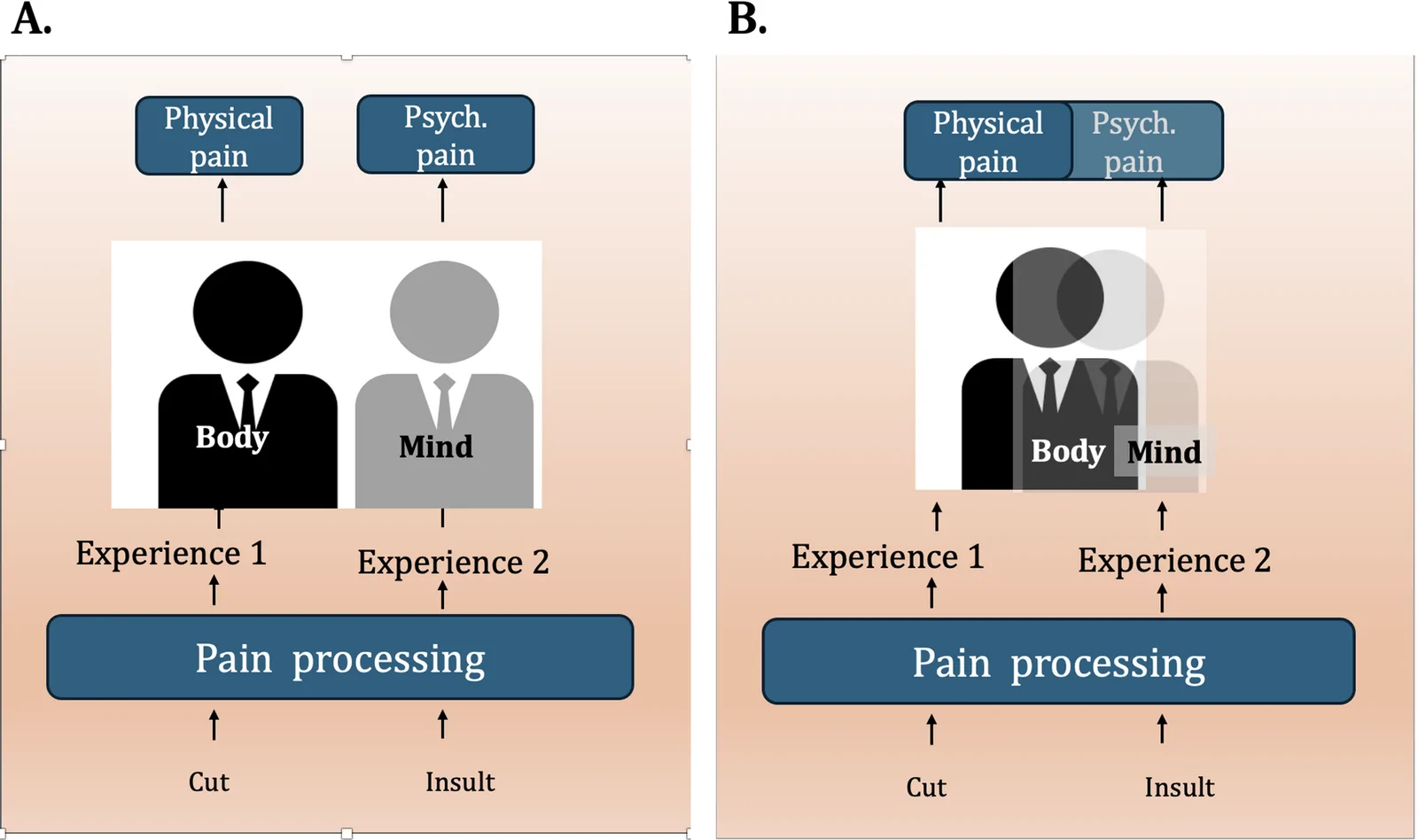
The putative role of intuitive Dualism in pain perception among people who do not engage in NSSI (i.e., Controls; **A**) and those who do (**B**). Dualism could guide the interpretation of experiences of noxious and psychological stimuli (e.g., cut vs. social insult, respectively) as physical vs. psychological pain. An attenuation of the mind-body divide via NSSI engagement could diminish the distinction between physical and psychological stimuli, and thus, blur the subjective experience of pain. The person image is from pixabay.com (https://pixabay.com/vectors/business-doctor-pharmacist-person-1316931/, accessed in May 2026, and modified by the authors). The image is used under the Pixabay license, which allows free use and adaptation of content.

Berent^13^ suggests that intuitive Dualism—the tacit view of the mind as ethereal, distinct from the body—could contribute to this distinction. If minds and bodies are contrasting conceptual categories, then psychological pain—an aspect of the mind—would naturally appear as utterly distinct from physical pain—an aspect of the body (Fig. 1A). A conceptual mind-body distinction would thus render physical and psychological pain as opposites, akin to how we naturally contrast objects and agents^14^. It is precisely because the neural signals of physical and psychological pain are interchangeable that their differentiation presents a pressing psychological problem.

In fact, this challenge facing pain recognition is but a special case of a broader challenge of emotion recognition. Affective science suggests that all emotional experiences are informed by internal bodily signals, which are highly ambiguous^15–17^. The signals for “anger” for instance, largely overlap with the physical state of “hunger”^18–20^. Difficulties with the interpretation of these bodily signals have been linked to psychopathology^18–20^, including NSSI^21^. By contrasting mind and body, intuitive Dualism prompts a distinction between emotions and physiological states (e.g., anger vs. hunger), and in so doing, it could play an adaptive role in emotion recognition, generally.

Critically, if Dualism typically promotes the segregation of physical and psychological pain in the recognition of affect (including psychological pain, a particular type of affect), then an attenuation of the perceived mind-body divide ought to reduce it, leading to affective dysfunction—a decrease in the ability to subjectively discern what one is feeling (Fig. 1B). For example, as a person experiences a distressing social interaction, they might cognitively note that “something went awfully awry”, but subjectively, at the emotional level, they might feel no emotion, and this contrast would be a source of distress. NSSI engagement could help interpret one’s internal signals by linking these signals with a conscious state (e.g., of physical pain), and in so doing, make sense of the distressing social situation. The conclusion—“it’s pain”—eliminates the affective distress associated with “feeling nothing”, reduces suffering, and incurs affective benefits.

At a broader level, our model suggests that individual differences in the mind-body divide could shed light on psychopathology. In line with this proposal, past research shows that Dualism varies systematically across various subgroups^22–24^, including clinical conditions^22,23^. In particular, autistic individuals exhibit an attenuation of the perceived mind-body divide^22^, whereas in transgender people, intuitive Dualism is augmented, and its magnitude is linked to their gender dysphoria^23^. Here, we explore whether an attenuation in intuitive Dualism is linked to NSSI.

### The present study

To gauge the role of Dualism in NSSI, we compare mind-body intuitions among people with a lifetime history of NSSI and Controls (i.e., people who have never engaged in NSSI). In the former group, we further assess the relationship between mind-body intuitions and self-reported motivations for NSSI engagement.

To evaluate mind-body intuitions, we asked participants to intuit whether a person’s psyche would persist in two tasks. The body-replication task invites people to imagine it were possible to create an exact duplicate of a person’s physical body. In the afterlife task, people are asked to assume that a person’s psyche could survive their physical death. The challenge, in both cases, is to indicate which features of the person’s psyche would persist. Two types of psychological traits are of interest: the person’s thoughts, such as forming words or beliefs in the supernatural—hereafter, *epistemic traits*, and the person’s emotions and actions (e.g., shame, squatting down), hereafter, *nonepistemic traits.*

Consider the predictions for the body-replication task. In the eyes of the Dualist, the psyche is distinct from the body, so simply copying one’s body will not necessarily preserve one’s psyche. Furthermore, for the Dualist, psychological traits may not all seem equally ethereal. Past research has shown that people can readily link actions (e.g., squatting down) and emotions (e.g., anger) with the physical body (e.g., with the legs and face, respectively)^25,26^, whereas thoughts seem devoid of a bodily anchor^26^. For this reason, a Dualist ought to consider nonepistemic traits (e.g., squatting down) as more likely to emerge in a body-replication than would epistemic traits (e.g., having a concept of time). A Physicalist, by contrast, would be more inclined to consider body and mind as one. Accordingly, for the Physicalist, duplicating one’s body ought to fully preserve all of one’s psychological traits—epistemic or not. If people with NSSI veer toward Physicalism, then (compared to Controls) the NSSI group should be more likely to endorse that the body-replication task would preserve the psyche.

To ensure that this group difference could not arise from a simple bias to respond “yes” (i.e., that all traits will emerge in the body replica), in a second task (administered to the same group of participants, counterbalanced for order), we asked participants to consider the converse—whether those same traits would persist in the afterlife, a situation that obliterates the body. If group differences arise from differences in mind-body intuitions, then now, the pattern of results ought to shift. For the Dualist, the obliteration of the body could preserve the psyche, so a Dualist ought to conclude that the psyche *could* persist in the afterlife, especially, epistemic traits (as those seem particularly ethereal). A Physicalist, by contrast, aligns the psyche with the body, so they should conclude that, once the body is no more, the psyche should also be no more. If NSSI indeed is associated with Physicalism, then, in the afterlife task, the NSSI group should now be less likely to respond “yes” (relative to Controls).

Altogether, then, we expect the NSSI group to veer towards Physicalism—that is, to view the psyche as more likely to persist in the replication task, and to desist in the afterlife. Thus, the NSSI group ought to show a stronger positive effect of task (∆Replication-Afterlife), evident as a steeper slope.

These effects could further depend on the severity of NSSI. If NSSI is associated with Physicalism, then people with a more severe lifetime history of NSSI (i.e., a greater number of lifetime NSSI episodes) ought to show stronger Physicalism.

To ensure that these differences are not due to autism (which is comorbid with NSSI^27^ and is marked by weaker Dualism)^25^, we excluded autistic participants from the sample. To further control for autistic traits, we entered eligible participants’ level of autistic traits (lower vs. higher, as assessed via the Autism-Spectrum Quotient [ASQ])^28^ into the main statistical models. We expected that the NSSI group would show stronger Physicalism even when autistic characteristics were controlled.

In our model, stronger Physicalism among the NSSI group may attenuate not only the differentiation of physical and psychological pain, but also emotion recognition generally; the higher level of alexithymia in individuals with NSSI is consistent with this proposal^29^. As a result, people who engage in NSSI might also struggle to identify and reason about their psychological suffering.

To explore this possibility, we asked participants who engage in NSSI to evaluate various statements concerning the functions of their self-injury. We expected that participants with weaker Dualism might be less likely to identify the functions of their NSSI.

## Methods

### Participants

**Fig. 2:**
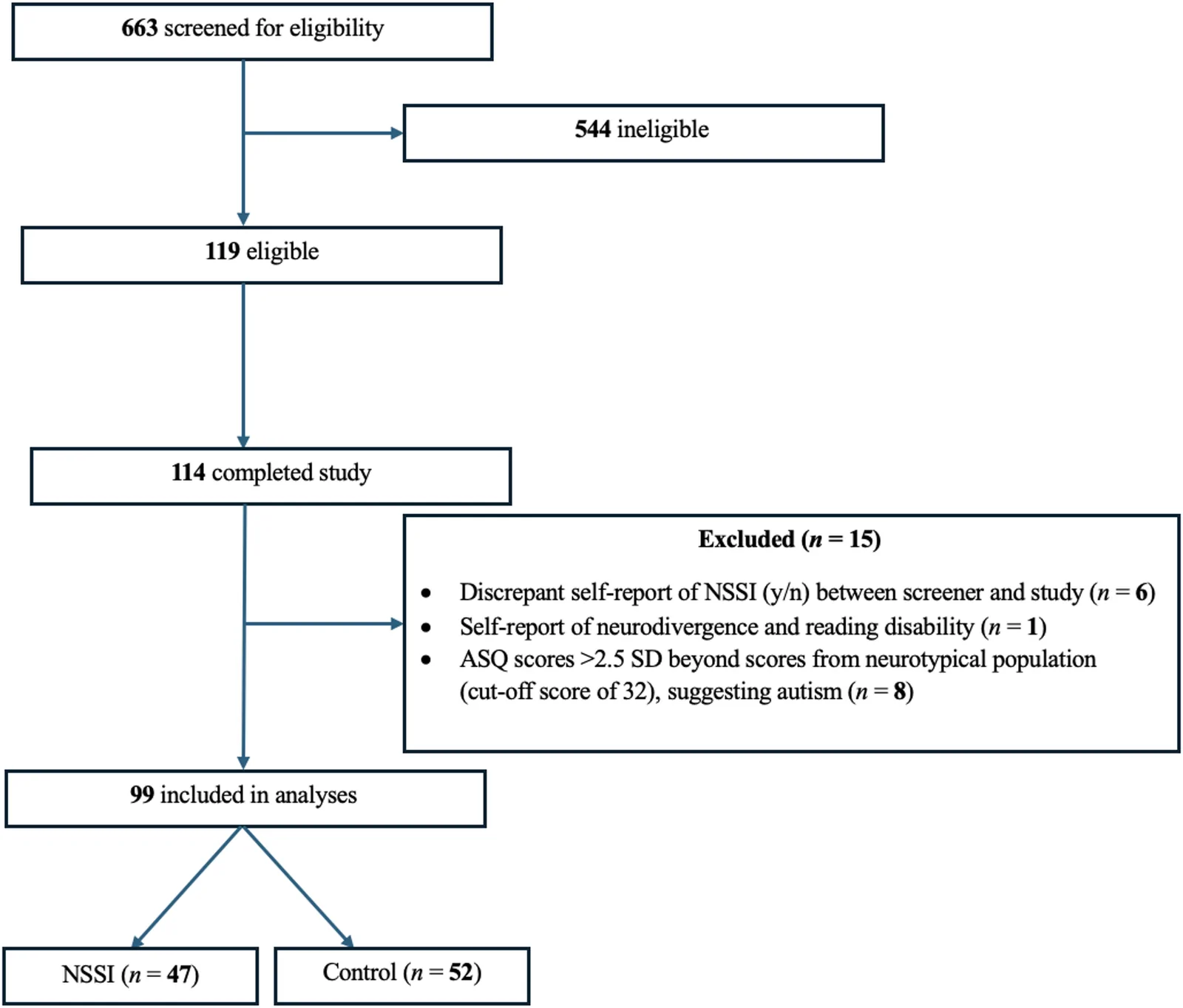
Eligibility flowchart, summarizing the recruitment, selection and exclusion of study participants.

Two groups of young adults (aged 18–40 years) were recruited for an online study. One group (*N* = 47) consisted of individuals who self-reported at least 5 lifetime NSSI episodes, with at least 1 occurring within the past 5 years; the other group consisted of individuals who reported never engaging in NSSI (Controls; *N* = 52). Participants’ eligibility was assessed via a pre-screener, administered prior to the experiment. The pre-screener, along with the full list of inclusion and exclusion criteria, is detailed in the SM. Figure 2 summarizes the screening and exclusion procedures.

In the absence of prior research on the role of intuitive Dualism in NSSI, we were unable to estimate the sample size using power analysis; as such, the present results should be considered as exploratory.

*Informed consent:* All materials and procedures were reviewed and approved by the Institutional Review Board at Harvard University; all methods were performed in accordance with their respective guidelines and regulations. Informed consent was obtained from all participants.

*Image use:* The person image is from pixabay.com (https://pixabay.com/vectors/business-doctor-pharmacist-person-1316931/), accessed in May 2026, and modified by the authors. The image is used under the Pixabay license, which allows free use and adaptation of content.

### Materials

The experimental session featured the following tasks (in this order): a Dualism task (including two behavioral sub-tasks), a Theory of Mind (ToM) task, the Autism Spectrum Questionnaire (ASQ), the Inventory of Statements About Self-Injury (ISAS) questionnaire, and a demographics survey. All materials are provided as appendices (see SM, Appendix I).

- *Dualism task*. The Dualism task (Appendix I, sections b–d) evaluated participants’ tacit intuitions about bodies and minds, using the materials and procedures validated extensively in previous published research^22–24^. To gauge mind-body intuitions, we presented participants with a list of 80 psychological traits. Half of those traits captured knowledge and beliefs (e.g., Having classification of animals; i.e., epistemic traits); the other half consisted of actions and emotions (e.g., sitting, walking; i.e., nonepistemic traits). Each participant was asked to determine the propensity of these traits to emerge in two hypothetical situations. The body-replication scenario invited participants to suppose it were possible to grow a replica of the body of an adult human donor. The replica preserves every aspect of the human body and brain. With this in mind, participants were asked to determine whether or not a given trait would likely emerge in the replica. In the afterlife scenario, participants were invited to judge whether each trait would persist in the afterlife. Participants were told that whether or not there is an afterlife is unknown. But for the purpose of this experiment, participants were asked to assume that after people die, they do continue to exist in some capacity. The task was to reason about which human traits would likely be maintained in the afterlife (for the full instructions, see SM, Appendix). In both tasks (replication and afterlife), “yes” responses (coded as 1) indicated that a given trait would emerge; “no” (coded as 0) indicated that it wouldn’t. Each task featured half of the traits, and each participant responded to the same set of traits in both tasks. Trait and the task order were counterbalanced across participants, and trait order was randomized.
- *Theory of Mind (ToM) Task*. In the ToM task (adapted from30 and used in previous research ^22^,^24^), participants were presented with 20 short vignettes (randomized for order) in which a protagonist has potentially acquired a certain belief. In half of the vignettes, the belief was false, and the protagonist was likely to hold that belief (e.g., When Lisa left Jacob, he was deep asleep on the beach. A few minutes later a wave woke him. Seeing Lisa was gone, Jacob decided to go swimming. Lisa now believes that Jacob is sleeping); the other described a true belief that the protagonist was unlikely to hold (e.g., Anne made lasagna in the blue dish. After Anne left, Ian came home and ate the lasagna. Then he filled the blue dish with spaghetti and replaced it in the fridge. Anne thinks the blue dish contains spaghetti). The probe sentence stated that the protagonist holds a certain mental state; participants were instructed to quickly determine whether that statement was true or false. For additional details, see SM; for the full materials and instructions, see Appendix I, sections e and f.
- *Autism Spectrum Questionnaire (ASQ)*. Given that autism spectrum disorders are often comorbid with NSSI31, and are further linked to intuitive Dualism22, we assessed autism spectrum characteristics using the ASQ (from28; Appendix I, section g).
- Inventory of Statements About Self-Injury (ISAS) questionnaire. The ISAS32(see SM, Appendix I, section h) was used to measure different aspects of NSSI, including age of onset, date of last episode, frequency of lifetime engagement across different methods (e.g., cutting, biting, burning; 12 methods assessed), and motivations for NSSI (e.g., affect regulation, self-punishment, peer-bonding; 13 motivations assessed). Additional items asked about time from urge-to-action, whether NSSI is done alone, physical pain experienced during NSSI, and desire to stop self-harm.
- Demographics survey. The demographics survey (Appendix I, section i) included items assessing participants’ age, sex at birth, gender identity, sexual orientation, race, education level, and employment status; their demographics are described in Table S1. ASQ characteristics of the NSSI group are described in Table S2.

## Results and discussion

The following analyses test two critical predictions of our model. First, in people who engage in NSSI, the mind-body divide may be attenuated. Second, this attenuation of the mind-body divide may impair the interpretation of one’s affective states, including those leading to NSSI engagement.

We assess each of these predictions in distinct sections. “Is intuitive Dualism attenuated in NSSI?” evaluates the first hypothesis via a mixed-effect logistic regression model. In this model, Physicalism is gauged by the slope of the Task factor: the more positive the slope (i.e., the more likely participants are to state that replicating the physical body will preserve the psyche (more than its elimination, in the afterlife)), the more Physicalist the participant.

To shed light on the link between mind-body intuitions and affective processing, “Is Physicalism associated with lower endorsement of self-reported NSSI functions?” explores correlations between mind-body intuitions and the perceived functions of NSSI within the NSSI group. Here, Physicalism is operationalized as a *Physicalism score*, captured by differential responses in the replication relative to the afterlife task (for details, see “Is Physicalism associated with lower endorsement of self-reported NSSI functions?”). We expected that Physicalist participants (those with a higher *Physicalism score*) might be less likely to identify the functions of their NSSI. While our tests do not directly gauge affective processing nor can correlations speak to causation, such results would be consistent with the possibility that Physicalism is linked to affective processing in NSSI.

### Is intuitive Dualism attenuated in NSSI?

To evaluate intuitive Dualism in NSSI, we submitted participants’ responses (the proportion of “will emerge” responses) to a mixed-effects model using the glmer function of the lme4 package in R (R Core Team, 2016, version 2024.04.1 + 748), R version 4.4.0 (2024-04-24)^33^. The model is as follows (for all R code, see SM, Appendix II):

*Response (yes (1)*,* no(0)) ~ Trait [Epistemic*, Nonepistemic] * Task [Replication, Afterlife] * NSSI severity [Lower,* Higher*, Control] * ASQ Level [Lower,* Higher] + [Trait | subject] + [1 | item])*

In this model, Task, Trait, NSSI severity, and ASQ Level are fixed effects (for descriptive statistics, see SM, Table S2); subject and item (i.e., questions in the Dualism task) are random effects. A positive effect of Task (Replication + 0.5; Afterlife, − 0.5) thus indicates a higher proportion of “yes” responses to the body-replication task. For Trait (Nonepistemic, + 0.5 vs. Epistemic, − 0.5), a positive effect indicates a higher proportion of “yes” responses for nonepistemic traits.

The two additional fixed effects in the model—NSSI severity and ASQ Level—capture participants’ characteristics. NSSI severity indicates the number of lifetime NSSI episodes (reported in the pre-screener); ASQ Levels are indicated by ASQ scores. For both factors—NSSI severity and ASQ Level—subgroup membership (Lower vs. Higher) is defined using a median split. For autistic characteristics (Lower: +0.5; Higher: − 0.5), a positive effect indicates a higher proportion of “yes” responses for the lower ASQ group (relative to the higher group). NSSI severity (Lower, Higher, Control) was coded using contrast coding, comparing Controls to the lower and higher severity levels of NSSI.

In light of this study’s novelty, the analyses were not pre-registered; the choice of this statistical model was informed by a series of analyses that systematically evaluated the model’s fit (see SM, Tables S6–S7). The chosen model (reported above) offered the best fit unambiguously, and results from this model converged successfully (bobyqa optimizer, convergence code = 0) with a maximum of 800,000 function evaluations and showed no evidence of singularity (isSingular = FALSE). Below, we detail the model’s results, along with confidence intervals (CI; all CIs are 95% Wald CIs).

The findings suggest that, overall, across groups, participants leaned towards Dualism. Dualist intuitions, however, were linked to both NSSI severity and the level of autistic traits. These conclusions are gleaned from four interactions, detailed below. Since our dataset is small (NSSI: *N* = 47 (Higher severity: *N* = 23; Lower severity: *N* = 24); Controls: *N* = 52), the present results should be viewed as *exploratory.*

**Fig. 3:**
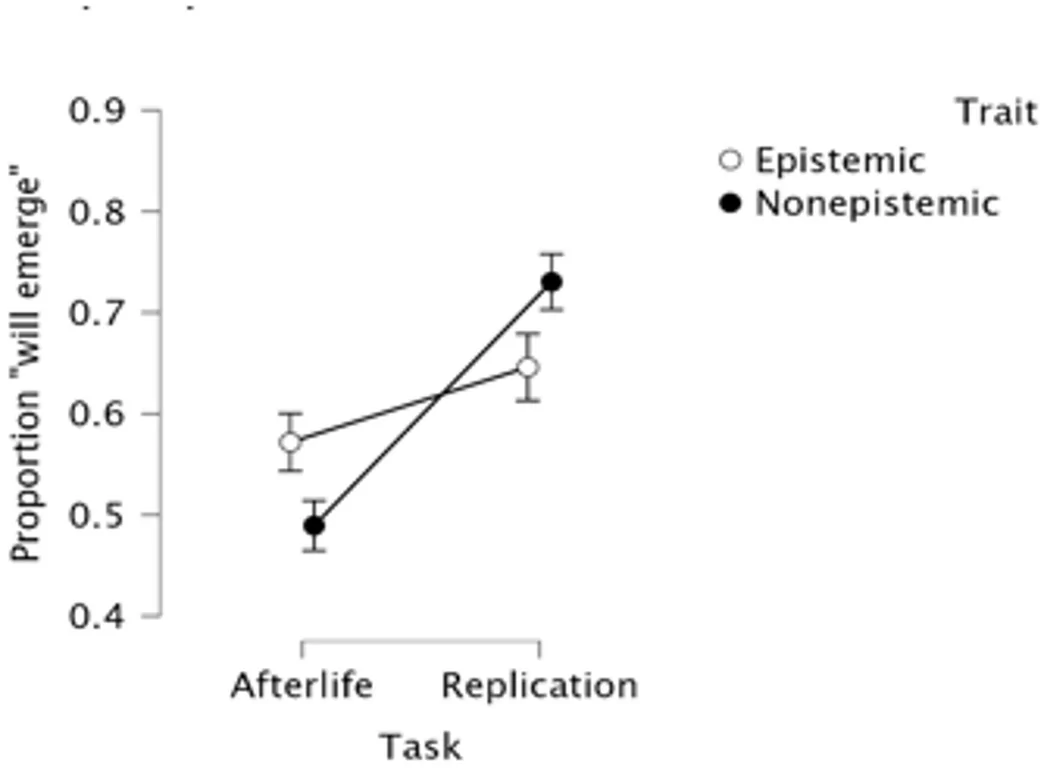
Mind-body intuitions (collapsed across NSSI and control groups). Mind-body intuitions are gauged by the interaction between task (afterlife vs. replication) and trait (epistemic vs. nonepistemic).
- *Severe NSSI is linked to mind-body intuitions.* The significant Task (Replication) x NSSI severity (Higher) interaction (β = 0.36, SE = 0.14, Z = 2.50, *p* = .012, CI: [0.079, 0.646]) indicated that mind-body intuitions were further modulated by the severity of NSSI, as participants with more frequent NSSI engagement (*N* = 23) showed a more pronounced effect of Task (i.e., they exhibited a steeper Task slope).
  Although all groups intuited that duplicating the body (in body-replication) would preserve the psyche (more than its obliteration, in the afterlife), this effect was especially pronounced in the group with more severe NSSI. This result suggests that participants with more severe NSSI view the psyche as more strongly anchored in the physical body, in line with Physicalism (Fig. 4A).
  The group with lower NSSI severity (*N* = 24), by contrast, showed a shallower effect of Task than Controls, resulting in a Task (Replication) x NSSI severity (Lower) interaction (β = − 0.49, SE = 0.14, Z = − 3.44, *p* < .001, CI: [− 0.765, − 0.210]). Thus, in the group with lower NSSI severity, the advantage of the replication task (as a means to preserve the psyche) was weaker than in the Controls. This result suggests that, unexpectedly, the lower severity NSSI subgroup slightly veered toward Dualism.
  This unanticipated result warrants attention because it questions the key finding that NSSI is linked to Physicalism. Accordingly, before considering the source of the findings from the group with lower NSSI severity, we first sought to ascertain whether the critical interaction between Task and NSSI severity is a reliable phenomenon, rather than an artifact of the median split procedure used to define severity.
  To this end, we conducted a sensitivity analysis to see whether results differed when participants’ NSSI severity was defined continuously, via NSSI lifetime engagement (log-transformed; LN([X + 1)). The full model was response *(yes (1)*,* no(0))* ~ Trait [Epistemic, Nonepistemic] * Task [Replication, Afterlife] * Lifetime NSSI + [Trait | subject] + [1 | item]). The Task x Lifetime NSSI interaction remained significant (β = 0.074, SE = 0.03, Z = 2.32, *p*=.02, CI [0.012, 0.136]; for the full results, see SM, “Is Physicalism associated with lower endorsement of self-reported NSSI functions?”). As in the omnibus model (with a categorical definition of NSSI severity), the continuous severity model showed that participants with more severe NSSI showed a steeper Task slope; this is in line with the hypothesis that NSSI is associated with stronger Physicalism.
  Having established that the interaction between NSSI severity and mind-body intuitions does not depend on the specific operationalization of severity, we return to our original puzzle: why do participants with lower NSSI severity show stronger Dualism than Controls (in the omnibus, categorical model)?
  It is unlikely that this effect arises from NSSI per se. Indeed, it is difficult to see why NSSI would attenuate Dualism when NSSI is severe and augment it when NSSI is less severe. It is also unlikely that these differences arise because the higher and lower NSSI severity groups differed on ToM or autistic characteristics (both of which have been associated with Dualism); for all factors, t < 1.5, *p*>.15 (a two-sample t-test, two-tailed).
  We thus consider two explanations for this finding. One possibility is that these differences arose because the lower severity NSSI group had a higher proportion of women (79%) relative to the Controls (69%); women are demonstrably more prone to Dualism (relative to men)^24^. To correct for this gender imbalance, we re-examined the effect of Task and NSSI severity for women only (which accounted for 71% of the participants in the total sample). The Task (Replication) × NSSI severity (Lower) interaction was now statistically non-significant (β = − 0.556, SE = 0.700, Z = − 0.794, *p* > .43, CI: [− 1.929, 0.817]; see Fig. 4B). In contrast, the evidence for the Task (Replication) × NSSI severity (Higher) interaction persisted, although it did not reach statistical significance (β = − 1.537, SE = 0.830, Z = − 1.851, *p* < .06, 95%, CI [− 3.165, 0.090]). This result suggests that the sex imbalance could have contributed to the stronger Dualism in the lower severity NSSI group.
  Another possibility (not inconsistent with the first) is that the higher and lower severity NSSI groups have different pathways to NSSI, and the attenuation of Dualism only plays a role in more severe NSSI. For example, it is possible that, in the lower severity group, NSSI arises primarily for social reasons, whereas in the higher severity group, affective processing (which, by hypothesis, is guided by Dualism) plays a greater role. An analysis of participants’ self-reported motivations for their NSSI (reported below; “Is Physicalism associated with lower endorsement of self-reported NSSI functions?”) is in line with this proposal.

- *Overall Dualism*. Across groups (NSSI and Controls), there was a significant effect of Task (Replication: β = 0.95, SE = 0.08, Z = 11.75, *p* < .001, CI: [0.792, 1.109]), such that the psyche seemed more likely to persist in the body-replication condition relative to the afterlife task. In other words, people considered the psyche to be more durable in a scenario that duplicated the body (body-replication) than in one that obliterated it (afterlife).
  This advantage of the body-replication task, however, was further modulated by trait, resulting in a Task (Replication) * Trait (Epistemic) interaction (β = 0.92, SE = 0.16, Z = 5.73, *p*< .001, CI: [0.607, 1.238]). This interaction emerged because the slope associated with the effect of Task was steeper for nonepistemic traits relative to epistemic traits. Figure 3 plots the results; this and all subsequent figures were generated in JASP^34^; error bars are SEs.
  Thus, when compared to the afterlife (a situation that obliterates the body), duplicating one’s body seemed better able to preserve one’s actions and emotions (i.e., nonepistemic traits) than one’s thoughts (i.e., epistemic traits) across the sample. This finding suggests that thoughts (epistemic traits) seem somewhat less firmly anchored in the body, in line with Dualism.

**Fig. 4:**
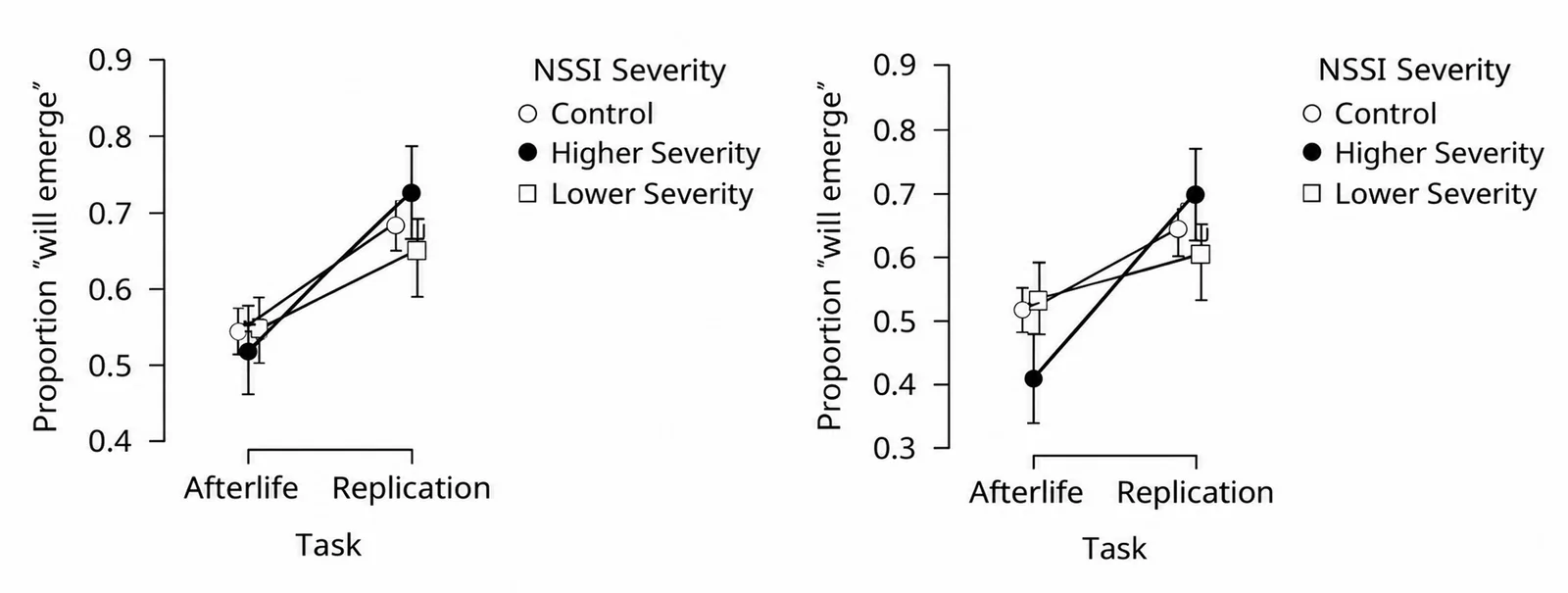
The modulation of mind-body intuitions by NSSI severity (higher severity, lower severity, and controls) across all participants (**A**) and women only (**B**). Mind-body intuitions are gleaned from the slope of Task (a larger slope suggests stronger Physicalism). NSSI severity is defined by a median split of lifetime NSSI episodes.
- *Autistic characteristics are linked to Physicalism*. The omnibus mixed-effects model further showed that autistic characteristics (captured by ASQ Level) modulated mind-body intuitions, as the Task (Replication) × Trait (Epistemic) × ASQ Level interaction was statistically significant (β = 0.64, SE = 0.32, Z = 2.01, *p* = .045, 95% CI [0.015, 1.269]). In individuals with stronger autistic characteristics (the higher ASQ Level: *N* = 48), the advantage of the body-replication (over the afterlife task) was stronger than in those with weaker autistic traits (in the lower ASQ Level, *N* = 51). Thus, the slope of Task was twice as large in individuals with higher ASQ (β = 1.24) compared to those with lower ASQ (β = 0.60).
  An inspection of Fig. 5, however, suggests that this group difference was especially pronounced for epistemic traits (Fig. 5A). This is only expected, as nonepistemic traits (e.g., squatting, anger) are typically perceived as inherently embodied (e.g., in the legs, facial expression)^22,24,35,36^. Accordingly, it is for the seemingly ethereal, epistemic traits (i.e., thoughts) that Physicalist tendencies ought to be more pronounced. These considerations explain why differences in the Physicalist intuitions (between the higher- and lower-level ASQ subgroups) emerged for epistemic traits.
  **Fig. 5:**
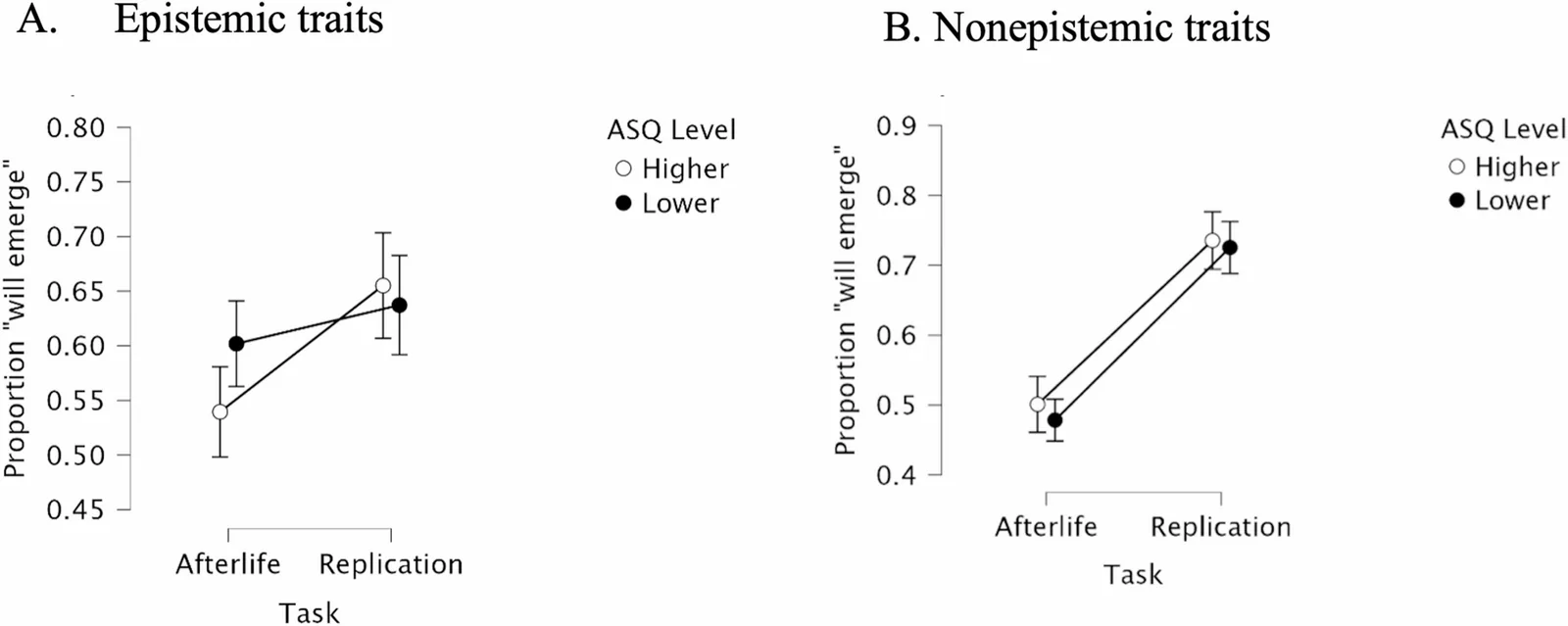
The modulation of mind-body intuitions by ASQ level for epistemic (**A**) and nonepistemic (**B**) traits. Mind-body intuitions are gauged by the slope of task (a larger slope suggests stronger physicalism). ASQ level is gauged by a median split on the ASQ scores (higher vs. lower scores).
  **Fig. 6:**
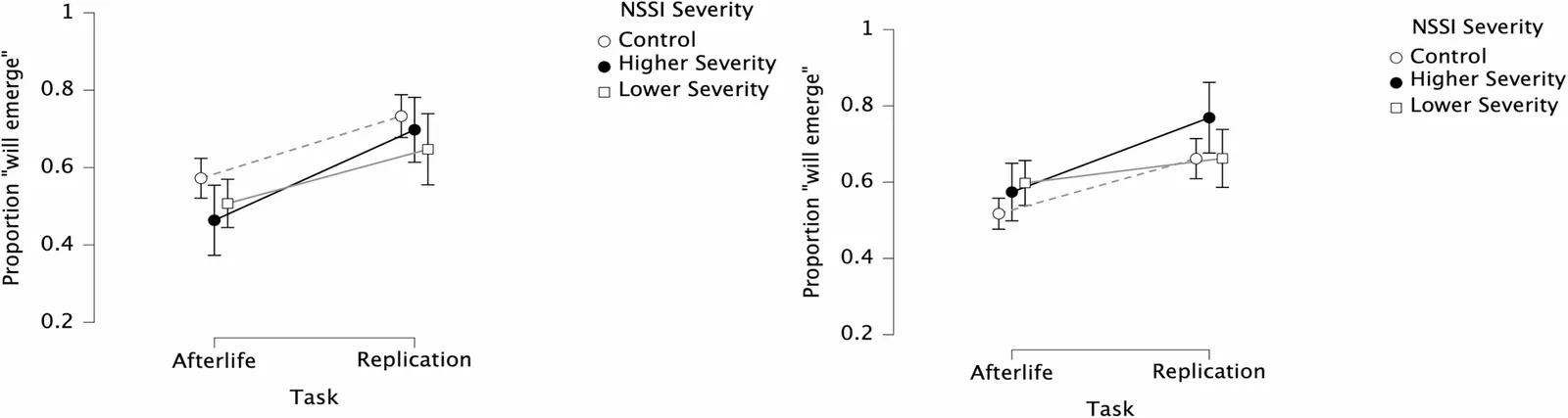
The modulation of mind-body intuitions by NSSI severity (higher, lower, controls) and ASQ level (lower: **A**; higher: **B**). Mind-body intuitions are gleaned from the slope of task (a steep slope suggests physicalism). NSSI severity is defined by a median split of lifetime NSSI episodes; the ASQ level is defined by a median split of the ASQ scores.
- *Physicalism is linked to more severe NSSI and to stronger autistic characteristics.* The regression model further showed a Task (Replication) × NSSI severity (Lower) × ASQ Level Interaction (β = − 0.76, SE = 0.28, Z = − 2.69, *p* = .007, CI: [− 1.318, − 0.206]). The interaction indicated that the effect of Task differed across the NSSI severity levels, depending on the level of autistic traits (ASQ Level).
  To interrogate this interaction, we examined the simple effect of Task (Replication vs. Afterlife) separately within each NSSI Severity × ASQ Level subgroup. Table 1 provides the slopes for each of the six subgroups, along with Standard Errors, the corresponding sample sizes, and CIs (slopes are estimated using the emmeans function in R).
  **Table 1:** Simple effect of task (replication vs. afterlife) across NSSI severity and ASQ level.

NSSI severity	Higher ASQ level				Lower ASQ level			
	β	SE	95% CI	*N*	β	SE	95% CI	*N*
Controls	0.97	0.13	[0.72, 1.21]	20	0.94	0.10	[0.74, 1.13]	32
Lower	0.86	0.15	[0.57, 1.15]	15	0.07	0.18	[− 0.29, 0.42]	9
Higher	1.49	0.15	[1.19, 1.79]	13	1.14	0.19	[0.77, 1.51]	10

*β* values represent the simple effect of Task (Replication vs. Afterlife) estimated from the generalized linear mixed model on the log-odds scale. Positive coefficients indicate greater endorsement of the Replication than the Afterlife task. Confidence intervals are 95% Wald intervals estimated from emmeans. Results are averaged across Trait levels (epistemic vs. nonepistemic).
  An inspection of the means suggests that the effect of Task was numerically stronger in the higher NSSI severity group than in Controls, regardless of ASQ Level. Additionally, within each NSSI level, the effect of Task was numerically stronger for participants with higher levels of autistic traits (relative to those with lower levels).
  Similar conclusions emerged when the group with higher NSSI severity was compared with the group with lower severity. The more severe level of NSSI was associated with a numerically stronger effect of Task. Additionally, the effect of Task was numerically stronger among participants with stronger autistic traits.
  Both findings are expected by our model. As noted, we view the steeper Task slope as evidence for Physicalism, and we expected stronger Physicalism to be associated with both NSSI severity and autistic characteristics^22^.
  Unexpectedly, however, we found that among participants with lower NSSI severity, those with stronger autistic characteristics showed a robust effect of Task (*b* = 0.86), whereas among those with weaker autistic characteristics, the effect of Task was virtually absent (*b* = 0.07). This contrasts with Controls (with a lower level of ASQ), who did show a robust effect of Task (*b* = 0.94).
  Given that this subgroup (participants with less severe NSSI and weaker autistic characteristics) is small (*N* = 9), the source of this finding is difficult to discern. One possibility is that this difference arose from the over-representation of women (in the lower ASQ-level, 78% compared to Controls, 58%); as noted, women independently lean towards Dualism^24^. Alternatively, participants with lower NSSI severity might have a partly different pathway to NSSI compared to those with more severe forms of NSSI. The correlational analyses reported next are consistent with this possibility.
- *Conclusions*. Summarizing, across groups, participants viewed thoughts as more ethereal, in line with Dualism. Participants with more severe NSSI, however, leaned towards Physicalism, as did participants with stronger autistic characteristics. Unexpectedly, the group with lower NSSI severity leaned towards Dualism, especially when their autistic characteristics were low. We suggest this effect arises, in part, from gender imbalance (as shown above, correcting the gender imbalance eliminated that effect). Another possibility is that our lower- and higher-NSSI severity groups reflect different NSSI trajectories. Either way, the results from the higherseverity subgroup tentatively suggest that more severe NSSI is associated with an attenuation in the tacit mind-body divide.

### Is physicalism associated with lower endorsement of self-reported NSSI functions?

Our theoretical model predicts that the putative attenuation of the mind-body divide in NSSI may disrupt affective processing. To explore this possibility, in the final section, we examine whether the severity of NSSI and mind-body intuitions are linked to participants’ self-identified functions of their NSSI (evaluated via the ISAS; for descriptive statistics, see Table S2). We hasten to note that a reduced endorsement of the ISAS factors could arise from many reasons unrelated to affective processing (e.g., participants rejecting the wording of specific items). As such, any links between ISAS functions and affective processing are *indirect*. Moreover, our correlational analysis only probes for associations between the endorsement of ISAS factors and Physicalism—it cannot speak to causation. Still, if the attenuation of the mind-body divide in NSSI is linked to affective dysfunction, then, in our NSSI group, Physicalist participants could conceivably be less likely to endorse the NSSI functions, and this should be especially likely when NSSI severity is higher.

To capture individual differences in mind-body intuitions, we computed a *Physicalism score* for each participant. To this end, we subtracted mean afterlife responses (across traits) from responses in the body-replication task (across traits). Thus, a positive ∆Task difference (i.e., higher “yes” response in the body-replication task) suggests that the psyche is perceived as embodied—as likely to persist when the body is copied (in the body-replication task) and to desist without it (in the afterlife task).

Next, we examined whether Physicalism scores are linked to self-reported functions of NSSI behavior. To tap into participants’ ability to reason about their NSSI generally (as opposed to endorsing any specific function, such as “self-care”), in this analysis, we chose to focus on two omnibus ISAS factors: Intrapersonal (e.g., affect regulation, anti-dissociation) and Interpersonal (e.g., peer bonding, toughness; for a detailed analysis of all 13 ISAS factors, see SM, Table S3). We expected that participants who veered toward Physicalism would be less likely to endorse these factors, and this would be especially the case when their NSSI was more severe.

To evaluate this prediction, we report these correlations by NSSI severity. We first report the correlations in the group with higher NSSI severity; we next inspect the group with lower NSSI severity and, finally, report the findings across all participants with NSSI.

All correlations are Spearman’s correlations, conducted with two-tailed tests in JASP^34^ (Pearson’s correlations could not be conducted soundly due to the presence of outliers). Spearman’s test assumptions were fully met. One outlier participant in the higher severity group was removed from the analysis (as their Physicalism and mindreading scores both fell 2.5 SD below the NSSI mean, suggesting inattentiveness). Thus, the correlational analyses are based on 46 NSSI participants (in the subgroup with higher NSSI severity: *N* = 22; in the subgroup with lower NSSI severity: *N* = 24).

**Fig. 7:**
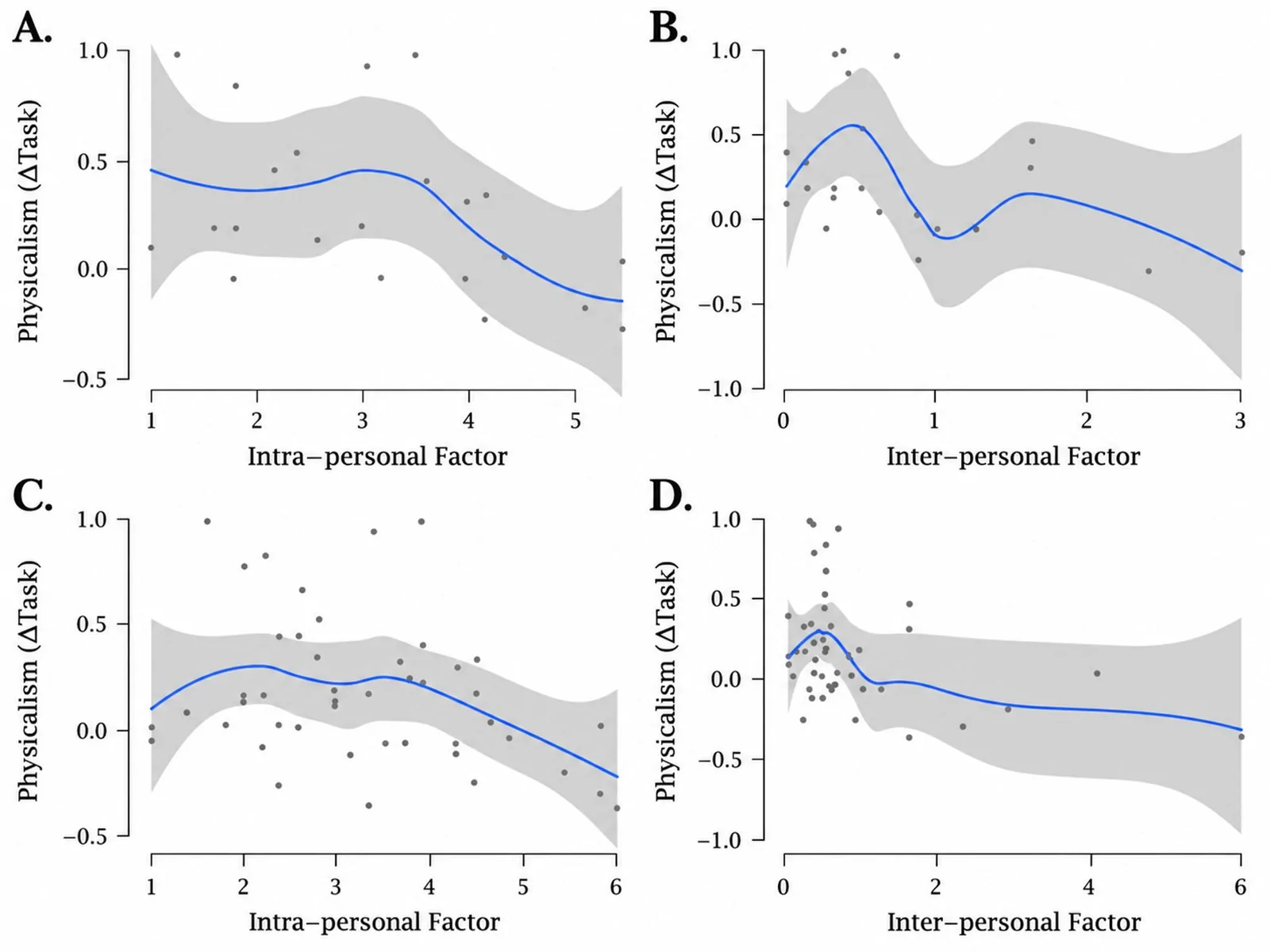
Simple correlations between Physicalism and the endorsement of the ISAS Intrapersonal and Interpersonal factors for participants in the higher NSSI subgroup (panels **A**,**B**) and for all participants with NSSI (panels **C**,**D**). NSSI severity (higher vs. lower) is defined by a median split of self-reported lifetime NSSI episodes.
- *Lower NSSI severity*. If the reluctance of Physicalist participants with severe NSSI to endorse the functions of their NSSI (as suggested by ISAS factors) arises, in part, from an affective dysfunction, then in less severe NSSI, one would expect the association with Physicalism to be attenuated. In line with these expectations, for lower-severity NSSI participants (*N* = 24), Physicalism did not correlate with either the Intrapersonal or Interpersonal factors, nor were the partial correlations significant (all *p* > .29).
- *Participants with NSSI engagement.* When considering the entire NSSI sample (*N* = 46), Physicalism was negatively correlated with both the Intrapersonal factor (r(44) = − 307, *p*=.038, Fisher’s Z = − 0.317) and the Interpersonal factor (r(44) = − 0.296, *p* = .045, Fisher’s Z = − 0.306). These correlations, however, were smaller than the correlations in the higher-severity group. Moreover, after controlling for multiple correlations (using BH-FDR method), only the correlation with the Interpersonal factor was significant (*p* = .045; for the Intrapersonal factor: *p* = .076). Finally, when the effects of mindreading and autistic traits were partialled out, neither of these correlations survived (for the Intrapersonal factor (r(41) = − 0.266, *p* = .084, Fisher’s Z = − 0.273); for the Interpersonal factor: (r(41) = − 0.283, *p* = .066, Fisher’s Z = − 0.291). The attenuated correlations in the full NSSI sample are expected, given that Physicalism was associated with ISAS endorsement among participants with higher NSSI severity, but not among those with lower severity.
- *Conclusions*. Altogether, the correlational analyses suggest a potential link between mind-body intuitions and participants’ reflections on their self-injury. The stronger the Physicalism, the less likely participants with higher NSSI severity were to endorse the ISAS Interpersonal and Intrapersonal factors as the motivations for their self-injury.
  Within the higher-severity subgroup, we found a negative association between the ISAS Interpersonal factor and Physicalism, which survived correction for individual differences in mind-reading and autistic characteristics. Moreover, the association between mind-body intuitions and the identification of NSSI functions was *selective* to the subgroup with higher NSSI severity; participants with lower NSSI severity did not show significant correlations between the endorsement of either Inter or Intrapersonal factors and Physicalism. This selectivity is predicted by our hypotheses that (a) the negative correlation (between NSSI and the endorsement of ISAS functions) arises, in part, from affective dysfunction, and (b) affective dysfunction is more prevalent in people with more severe NSSI.
  This conclusion, however, remains highly speculative, as the present study does not address affective dysfunction or affective processing, and the endorsement of the ISAS factors addresses affective processing rather indirectly. Other reasons for caution are presented by the small sample size and the failure of some findings to survive corrections for multiple comparisons. Future research is necessary to extend and replicate these findings. At present, we tentatively note that the negative association between Physicalism and self-reported motivations for self-injury is *consistent* with our proposal that Physicalism is associated with lower endorsement of standard self-reported explanations for self-injury.

- *Higher NSSI severity*. Analysis of the group with higher NSSI severity (*N* = 22) showed that as participants veered toward Physicalism (i.e., as they were more likely to state that the psyche would persist if the body were copied but not persist without the body as in the afterlife), they were less likely to endorse the ISAS Intrapersonal factor as an explanation for their NSSI (r(20)= − 0.475, *p* = .026, Fisher’s Z = − 0.516). A similar trend emerged for the Interpersonal factor (r(20) = − 0.420, *p* = .052, Fisher’s Z = − 0.448; see Fig. 7).
  When correcting for multiple correlations using Benjamini–Hochberg False Discovery Rate (BH FDR, with m = 2 tests per family), however, the correlation with the Intrapersonal factor was no longer significant (adjusted *p* = .052). Moreover, after adjusting for autistic traits and mindreading, the association between the Intrapersonal factor and Physicalism was further attenuated and was non-significant after the BH FDR adjustment (r(17) = − 0.42, adjusted *p* = .074, Fisher’s Z = − 0.447).
  For the Interpersonal factor, by contrast, the negative correlation with Physicalism score was *strengthened* when partialling out the effects of these covariates (autistic traits and mind-reading: r(17) = − 0.550, *p* = .015, Fisher’s Z = − 0.618), and this partial correlation remained significant after adjusting for multiple correlations (BH FDR, with m = 2 tests per family: *p*=.03). Altogether, then, these results suggest that as participants with higher NSSI severity veered towards Physicalism, they tended to be less likely to endorse Interpersonal functions for their NSSI, and showed a similar trend for Intrapersonal functions.
  As noted above, however, these correlations were obtained after one member of the higher-severity group was removed, and auxiliary analyses suggested that, once this individual was retained, the negative partial correlation with the Interpersonal factor was no longer significant. Given the small sample size of this subgroup (*N* = 22), one might therefore worry that these conclusions could be unstable. To evaluate this possibility, we examined the partial correlation after removing each of the remaining 21 participants, one participant at a time; 20 of these correlations were significant, and for the remaining one, *p*=.052 (for results, see SM, Table S5). These results suggest that the unique, negative association between the tendency toward higher NSSI severity and the endorsement of the interpersonal factor is stable within this sample.

## General discussion

NSSI presents an intriguing mind-body conundrum. Why do some individuals feel that the self-infliction of physical pain alleviates their psychological suffering?

We suggest that intuitive Dualism can shed light on the subjective experience of NSSI. We hypothesize that intuitive Dualism plays an adaptive role in typical emotion recognition, as it renders emotions, such as psychological pain, as distinct from physical states, such as noxious pain^13^. We further suggest that, in individuals who engage in NSSI, the perceived mind-body divide is attenuated. Consequently, these individuals struggle to recognize their psychological pain, and these challenges might further exacerbate their suffering. NSSI could help embody suffering and, for this reason, confer affective benefits. The results of this exploratory study offer preliminary support for this hypothesis.

### Main findings and their interpretations

Our study yielded three main findings. Below, we summarize the results and provide our interpretation of these findings.

First, participants intuited that duplicating a person’s body is less likely to preserve their thoughts (i.e., epistemic states) than their emotions and actions (i.e., nonepistemic states). We thus conclude that people tend to view thoughts as relatively disembodied and ethereal, in line with Dualism.

Second, we found that mind-body intuitions are modulated by the severity of NSSI. While all participants, on average, intuited that the psyche is more likely to persist by duplicating the body (in the body replication task) than by its obliteration (in the afterlife task), this intuition was stronger among people with a more severe lifetime history of NSSI (compared to those with lower NSSI severity and Controls). We interpret this result to suggest that individuals with more severe lifetime NSSI are inclined to view bodies and minds alike, in line with Physicalism. This finding is consistent with the hypothesis that the perceived mind-body divide is attenuated among people who engage in more frequent NSSI.

Surprisingly, participants with less severe NSSI were slightly *less* likely than Controls to state that duplicating a person’s body (in the body replication task) would preserve their psyche (relative to the psyche’s obliteration, in the afterlife), and this was especially the case when their autistic characteristics were weaker. This finding seems to suggest that, contrary to our hypothesis, participants with less severe NSSI veered toward Dualism.

This unexpected result might have arisen from a gender imbalance. In our sample, the subgroup with less severe NSSI included more women (relative to Controls), and past research has shown that women tend to be more Dualist than men^24^. Indeed, when adjusting for gender effects, this anomalous finding vanished: there was no longer a difference between the subgroup with lower NSSI severity and Controls—whereas the higher severity NSSI group still showed a Physicalist tendency (compared with Controls). Altogether, our results agree with the hypothesis that, among people with more severe NSSI, the perceived mind-body divide is attenuated.

Third, we found that mind-body intuitions correlated with participants’ likelihood of endorsing the ISAS factors as motivations for their NSSI behavior, and this correlation further varied with the severity of their NSSI. Specifically, among participants with more severe NSSI, the stronger the Physicalism (i.e., the more likely they were to state that duplicating the body, in the body-replication task, is more likely to preserve the mind than its obliteration, in the afterlife), the less likely they were to endorse ISAS functions for their NSSI, and this was especially the case for interpersonal NSSI functions. As noted, this correlation could arise for multiple reasons unrelated to affective processing (e.g., participants may fail to endorse certain ISAS factors because they disagree with the wording of these items). Nonetheless, our analyses suggest that this association is not due to explanations such as difficulties with mindreading or autistic traits and is specific to participants with more severe NSSI. We thus tentatively suggest that these correlational results are *consistent* with the possibility that NSSI severity is associated with stronger Physicalism. This in turn, may attenuate the relevance of the items contained on the ISAS as motivations for their NSSI.

### NSSI, Dualism and affective processing

Taken as a whole, the present exploratory study provides preliminary evidence that (a) the mind-body intuitions of people who engage in NSSI differ from those of Controls, and (b) mind-body intuitions may be linked to participants’ reflection on the function of their NSSI engagement. These conclusions converge with the existing literature linking NSSI to interoception difficulties^21^, alexithymia (i.e., difficulty identifying and describing emotions)^37,38^, affective dysregulation^39^, and pain-offset relief^6,40^. Mind-body intuitions outline a putative psychological mechanism that could connect these multiple proposals.

Berent^13^ hypothesizes that the perceived mind-body divide could contribute to the interoception of psychological states from internal bodily signals. As noted in the Introduction, one major challenge to interoception is that internal bodily signals are inherently ambiguous. For example, the physiological signals of anger are not inherently distinct from those that signal hunger; indeed, people systematically confuse the two^41–43^, as they do for noxious and psychological pain^12,44,45^. The mind-body divide could help alleviate the ambiguity by offering an extra “safety valve” that could aid interoception^13^. If, upon interoception, people would re-inspect their interpretation of physiological signals and tacitly evaluate whether they signal “mind” (e.g., anger, psychological pain) or “body” (e.g., hunger, physical pain), interoception accuracy is likely to improve, as is alexithymia.

From this model^13^, it follows that disruption of the mind-body divide may attenuate interoception, alexithymia, and affective processing generally, as well as the pain offset response in particular. Our proposal, then, offers a novel mechanism that could help explain how interoception works. Specifically, we suggest that the mind-body divide guides the interpretation of physiological signals of affect and, in so doing, improves emotional acuity. An attenuation of the mind-body divide could thus disrupt interoception, attenuate emotional acuity, and increase risk for clinical problems such as NSSI engagement.

We hasten to underscore that this proposal is strictly speculative at present. There is currently no evidence that Dualism mediates the relationship between interoception and affective processing. Moreover, the links between NSSI and Dualism in the present study are subject to several limitations. Below, we outline these shortcomings and note how they might be addressed by future research.

### Limitations and future directions

Several limitations of this study warrant consideration. First, our results are based on a single exploratory study. The sample size is also relatively small and involves even smaller subgroups of NSSI severity (i.e., more and less severe).

A second set of limitations concerns the characterization of the NSSI group. Information on NSSI severity was obtained entirely from online self-reports and so is potentially influenced by memory recall difficulties or biases. Moreover, because NSSI severity was defined by a median split of participants’ lifetime history of NSSI (as reported by participants), our severity measure is highly sample-specific and not generalizable to NSSI samples more broadly. The specific age range (18–40 years), language (English), and source of our participants (US-based Prolific workers) further limit the generalizability of our findings. In addition, in the absence of information about participants’ comorbid conditions (e.g., depression and anxiety, which are known comorbidities of NSSI)^46,47^, we presently cannot rule out the possibility that group differences in NSSI and its link to intuitive Dualism arise not from NSSI specifically, but rather from these comorbidities.

Another limitation concerns our experimental manipulations. Reasoning about hypothetical scenarios arguably offers an imperfect measure of Dualism. Additionally, we posit that Dualism affects emotion recognition in NSSI (including psychological pain), but our experiment does not directly assess emotion recognition or related constructs (alexithymia, interoception, dissociation, or pain sensitivity). Instead, we used the ability to identify self-reported functions of NSSI (via ISAS) as a proxy for emotion recognition, and we performed this inquiry in an exploratory manner, post hoc. Our conclusions are thus limited by the lack of a direct assessment or of a link between Dualism and affective processing. The reliance on multiple comparisons further limits the statistical robustness of the links between Dualism and the putative (indirect) measures of affect.

Finally, and critically, our results only suggest a correlation between Dualism and severe NSSI—they cannot establish causation, nor can they elucidate its direction: whether Physicalism is a cause of NSSI or its consequence remains unknown. In the theoretical model we have outlined (above), Physicalism may promote NSSI by impairing affective processing and exacerbating distress; physical pain helps people grasp their latent psychological anguish and confers affective benefits. However, the causal link could also go in the opposite direction: given that NSSI—an act involving physical pain to the body—alleviates mental anguish, this experience could lead people to view mind and body as linked. In this latter explanation, Dualism is a consequence of NSSI, rather than its cause. Relatedly, our study cannot determine whether the observed links between NSSI and Dualism are specific to NSSI or general, including its many comorbidities (e.g., depression and anxiety)^46,47^. Future research is needed to replicate and extend these conclusions to a larger sample using additional measures of Dualism (e.g., via implicit associations)^48^, tighter control of comorbidities, and direct assessment of affective processing.

Although our study leaves several critical questions unanswered, its results are noteworthy as they are the first to link NSSI behavior to tacit intuitions about minds and bodies. More generally, these findings converge with our previous results concerning the role of Dualism in gender dysphoria^23^ and autism^22^ to suggest that tacit, mind-body intuitions can shed light on the conscious self in both health and pathology.

## Supplementary Information

Below is the link to the electronic supplementary material.

Supplementary Material 1

## Data Availability

Upon publication, de-identified data and analysis scripts will be deposited in the OSF, subject to IRB approval. Should IRB approval for data deposition not be granted, data will be made available to qualified researchers under an appropriate data-use agreement that protects participant confidentiality.
