# Supplementary material for: Non-suicidal self-injury is linked to mind-body intuitions: an exploratory study
Source: Sci Rep. 2026 Oct 22;16:33393. doi: 10.1038/s41598-026-73035-3 (PMC13690570; doi:10.1038/s41598-026-73035-3)
Supplement: Supplementary file 1 [file 41598_2026_73035_MOESM1_ESM.doc]

**Non-suicidal self-injury is linked to mind-body intuitions:**

**An exploratory study**

**Supplementary Materials**

1. Methods
   1.1. Participants

Eligibility criteria for all participants included being a young adult (18-40 years old), being a native English speaker, residing in the United States, having normal-to-corrected vision, having no language or reading disorder, and having no formal diagnosis of autism spectrum disorder.

To be eligible for the non-suicidal self-injury (NSSI) group, participants were additionally required to report engaging in at least five lifetime NSSI episodes, with at least one NSSI episode occurring within the past five years. These criteria enabled us to focus on participants who engaged in NSSI recurrently and relatively recently. Additionally, NSSI and control groups were matched on biological sex to adjust for any potential confounds arising from sex-related differences in Dualism beliefs. Matching was achieved by first recruiting participants who engage in NSSI, then recruiting an equal number of male and female Control participants.

Participant eligibility was assessed via a screener administered to Prolific workers (see “pre-screener” below). Before administering the pre-screener, recruitment specifications were set within Prolific to reach our target population. Specifically, we used pre-screen items from Prolific to invite participants who were listed as living in the U.S., fluent in English, with normal-to-corrected vision, and without a diagnosis of dyslexia or autism spectrum disorder. Eligible participants, confirmed by our separate pre-screener, were then invited to participate in the experiment.

To be included in analyses, participants who completed the experiment were required to correctly respond to at least two out of three attention checks administered throughout the study (e.g., “please select slightly disagree for this item”). We further removed from analyses any participant whose self-report of lifetime NSSI engagement (y/n) in the screener was discrepant with their self-report of lifetime NSSI engagement (y/n) in the study (6 participants), as well as one participant who self-reported neurodivergence and reading disability (which is excluded by our eligibility criteria above). Eight additional participants were excluded because their Autism Spectrum Questionnaire (ASQ) scores were over 2.5 SD beyond scores from the neurotypical population (i.e., a cutoff of 32 on the ASQ^1^), suggesting autism. After participant removal, our dataset contained 99 participants, including 47 participants who engaged in NSSI (35 females, 12 males) and 52 controls (36 females, 16 males). Their demographic characteristics are shown in Table S1; the ASQ characteristics of our participants (Controls and NSSI of lower vs. higher severity) are summarized in Table S2.

Within this sample, 22 participants reported NSSI engagement in the past month (for the mean number of reported incidents: M=1.23, SD=2.12), 36 in the past year (M=7.06, SD=16.53), 43 in the past three years (M=17.94, SD=34.89), and all 47 reported NSSI engagement in the past five years (M=10.23, SD=16.44). The mean number of reported lifetime NSSI attempts was 49.09 (SD=76.75).

**Table S1. *Demographic Characteristics by Group***

| Characteristic | Control  (*n* = 52) | NSSI  (*n* = 47) |
| --- | --- | --- |
| Sex at birth (*n, %*) |  |  |
| Female | 36 (69.23%) | 35 (74.47%) |
| Male | 16 (30.77%) | 12 (25.53%) |
| Gender identity (*n, %*) |  |  |
| Woman | 36 (69.23%) | 35 (74.47%) |
| Man | 15 (28.85%) | 11 (23.40%) |
| Transgender/Non-binary | 1 (1.92%) | 1 (2.13%) |
| Race/ethnicity (*n, %*) |  |  |
| Asian | 5 (9.62%) | 2 (4.25%) |
| Black/African American | 11 (21.15%) | 6 (12.77%) |
| White | 31 (59.62%) | 34 (72.34%) |
| Hispanic/Latinx | 1 (1.92%) | 1 (2.13%) |
| Native American / Alaska Native | 0 (0.00%) | 1 (2.13%) |
| More than one | 4 (7.69%) | 3 (6.38%) |
| Sexual orientation (*n, %*) |  |  |
| Asexual | 1 (1.92%) | 1 (2.13%) |
| Bisexual | 3 (5.77%) | 13 (27.66%) |
| Heterosexual/Straight | 46 (88.46%) | 29 (61.70%) |
| Homosexual/Gay/Lesbian | 1 (1.92%) | 0 (0.00%) |
| Pansexual | 1 (1.92%) | 1 (2.13%) |
| Other | 0 (0.00%) | 1 (2.13%) |
| More than one | 0 (0.00%) | 2 (4.25%) |
| Education level (*n, %*) |  |  |
| Some high school | 1 (1.92%) | 0 (0.00%) |
| High school degree (or equivalent) | 18 (34.62%) | 10 (21.28%) |
| 2-year college degree / Associate’s | 3 (5.77%) | 6 (12.77%) |
| 4-year college degree / Bachelor’s | 16 (30.77%) | 27 (57.44%) |
| Master’s degree | 13 (25.00%) | 3 (6.38%) |
| Professional degree (e.g., MD, PhD) | 1 (1.92%) | 1 (2.13%) |
|  | *M SD* | *M SD* |
| Age | 29.90 5.60 | 29.50 5.76 |

*Note.* NSSI = Non-suicidal self-injury. Participants were able to select more than one response (as relevant) for “Race/ethnicity” and “Sexual orientation”. For each category, participants who selected more than 1 response are classified here as “More than one”.

***Table S2.*** *ASQ characteristics by group.*

| **Group** | **Subgroup** | **Mean** | **SD** | **Count** | **Min.** | **Max** |
| --- | --- | --- | --- | --- | --- | --- |
| **Control** | N/A | 20.62 | 4.61 | 52 | 11 | 29 |
| **NSSI** | All | 21.45 | 5.67 | 47 | 6 | 31 |
|  | Less severe | 21.46 | 4.84 | 24 | 11 | 30 |
|  | More severe | 21.43 | 6.54 | 23 | 6 | 31 |

*Note.* NSSI = ‘Non-suicidal self-injury’ ; ASQ= Autism-Spectrum Quotient; SD=Standard Deviation; Min=minimum; Max=Maximum.

1.2. Tasks and Procedure

In what follows, we provide additional details regarding the pre-screening task, experimental tasks, and procedure.

**Pre-screener**. Prospective participants (*N* = 663) completed a pre-screener (Appendix I, section a) via a Qualtrics link embedded in the Prolific post. Specifically, participants were invited to complete a pre-screener to see if they qualified for the “Beliefs About Mind and Bodies” study, which was advertised as focusing on how people think about minds and bodies. Screening occurred from June to August 2024. The pre-screener contained items asking about NSSI, including whether the participant ever engaged in NSSI, as well as participants’ estimated frequency of NSSI episodes in their lifetimes, in the past 5 years, in the past year, and in the past month. Consistent with previous NSSI research (e.g., ^2^), the pre-screener also asked about indirect forms of self-injury (drug and alcohol misuse, disordered eating) to reduce the likelihood that participants would identify the study aims. Additionally, the pre-screener included items assessing broader eligibility criteria (e.g., age, geographical location).

***Theory of Mind (ToM) Task*.** These materials were adapted from Dodell-Feder et al., 2011^3^, so as to dissociate the presence of true/false belief with its veracity. That is, in half of the vignettes, the belief was false, and the protagonist was likely to hold that belief (e.g., *When Lisa left Jacob, he was deep asleep on the beach. A few minutes later a wave woke him. Seeing Lisa was gone, Jacob decided to go swimming. Lisa now believes that Jacob is sleeping*), whereas in the others, the belief was true, but the protagonist was unlikely to hold it (e.g., *Anne made lasagna in the blue dish. After Anne left, Ian came home and ate the lasagna. Then he filled the blue dish with spaghetti and replaced it in the fridge. Anne thinks the blue dish contains spaghetti*). The probe sentence stated that the protagonist holds a certain mental state; participants were instructed to quickly determine whether that statement was true or false. These revised materials have been used in past research^4,5^ and have been shown to correlate with Dualist intuitions (for the full materials and instructions, see Appendix I, sections e and f). The order of the 20 vignettes was randomized.

Procedures. Participants were tested online, using a Qualtrics survey. Participants were first given the screener; after their eligibility was confirmed, they were invited to take part in the full study. In the main study, participants were first given the Dualism task (body-replication and afterlife tasks; counterbalanced within study groups), followed by the theory of mind (ToM) task, the ASQ, the Inventory of Statements about Self-Injury (ISAS), and a demographics survey. Participants who first completed the afterlife task (i.e., before the body-replication task) had a median completion time of 25 minutes and an average compensation of $14.72 per hour (summary statistics recorded in Prolific). Similarly, participants who completed the body-replication task first (i.e., before the afterlife task) had a median study completion time of 25 minutes and an average compensation of $14.34 per hour. After study completion, participants were provided a debriefing form outlining study objectives. A list of treatment resources (e.g., the 988 hotline) was also provided to all participants.

2. Correlational results

In what follows, we provide additional information concerning the correlational analyses reported in the main text (Section 3.2). These analyses examine whether participants’ self-reported motivations for their NSSI varied by their mind-body intuitions and by the severity of their NSSI. Subjective motivations for NSSI were evaluated via the ISAS.

2.1. Descriptive statistics

Table S3 provides descriptive statistics of all NSSI participants (N=47), stratified by the severity of NSSI (defined by a median split: Lower NSSI Severity: *n*=24; Higher NSSI Severity: *n*=23; the maximum score on any ISAS function is 6). A series of independent-samples t-tests (two-tailed) showed that NSSI severity did not modulate endorsement of any ISAS function (all p > .29).

**Table S3. Self-reported motivations for NSSI.**

|  | Lower NSSI Severity | | Higher NSSI Severity | |
| --- | --- | --- | --- | --- |
| ISAS Function | Mean | SD | Mean | SD |
| Affect regulation | 4.25 | 1.73 | 4.17 | 1.8 |
| Interpersonal boundaries | 0.92 | 1.64 | 0.7 | 1.3 |
| Self-punishment | 3.13 | 2.23 | 3.91 | 2.19 |
| Self-care | 1.38 | 1.81 | 1 | 1.6 |
| Anti-dissociation | 2.54 | 1.87 | 2.52 | 1.97 |
| Anti-suicide | 1.88 | 2.01 | 2.04 | 2.12 |
| Sensation seeking | 0.79 | 1.77 | 0.7 | 1.11 |
| Peer bonding | 0.29 | 1.23 | 0 | 0 |
| Interpersonal influence | 0.92 | 1.61 | 1.09 | 1.38 |
| Toughness | 1.33 | 1.66 | 1.22 | 1.48 |
| Marking distress | 2.29 | 2.33 | 2.61 | 1.85 |
| Revenge | 0.58 | 1.47 | 0.78 | 1.54 |
| Autonomy | 0.5 | 1.69 | 0.43 | 0.84 |
| Intrapersonal factor | 2.82 | 1.36 | 3.05 | 1.46 |
| Interpersonal factor | 0.84 | 1.37 | 0.74 | 0.78 |

Note: SD = Standard Deviation. The Interpersonal factor is the mean of autonomy, interpersonal boundaries, interpersonal influence, peer-bonding, revenge, self-care, sensation seeking, and toughness functions; The Intrapersonal factor is the mean of affect regulation, self-punishment, anti-dissociation / feeling generation, anti-suicide, and marking distress functions.

2.2. Correlations with specific ISAS functions

To determine whether participants’ motivation for NSSI varies by their mind-body intuitions, we examined whether Physicalism scores (∆Task, as defined in the main text) are linked to self-reported motivations for NSSI behavior. As noted in the main text, one participant with higher NSSI severity was identified as an outlier and removed from all analyses, so the correlational analyses are based on 22 higher-severity participants and 24 lower-severity ones.

Table S4 provides the simple Spearman’s correlations between Physicalist scores and the ISAS functions, as well as the partial Spearman’s correlations (controlling for ASQ and mindreading). An inspection of the simple correlations (Table S4) showed that, in the higher severity subgroup, there was a significant negative association between Physicalism and self-care, and this correlation remained significant after controlling for autistic properties and mindreading: the more strongly participants veered toward Physicalism, the less likely they were to attribute their NSSI to self-care (e.g., “giving myself a way to care for myself (by attending to the wound)”).

**Table S4.** Spearman’s correlations (simple and partial) between Physicalism and ISAS scores

|  | **Simple correlations** | | **Partial correlations** | |
| --- | --- | --- | --- | --- |
|  | **NSSI Severity** | | **NSSI Severity** | |
|  | **Lower** | **Higher** | **Lower** | **Higher** |
| Affect regulation | 0.344 | -0.34 | -0.131 | -0.398 |
| Interpersonal boundaries | 0.216 | -0.304 | 0.056 | -0.378 |
| Self-punishment | 0.428* | -0.364 | 0.067 | -0.183 |
| Self-care | 0.339 | -0.433* | -0.016 | -0.508* |
| Anti-dissociation | -0.01 | -0.243 | -0.214 | -0.204 |
| Anti-suicide | 0.387 | -0.416 | 0.040 | -0.374 |
| Sensation seeking | 0.133 | -0.146 | 0.134 | -0.154 |
| Peer bonding | 0.179 | NA | 0.080 | NA |
| Interpersonal influence | 0.416* | -0.329 | -0.065 | -0.320 |
| Toughness | 0.613** | -0.33 | -0.369 | -0.269 |
| Marking distress | 0.29 | -0.309 | -0.207 | -0.342 |
| Revenge | 0.339 | 0.061 | -0.275 | -0.255 |
| Autonomy | -0.107 | -0.261 | -0.191 | -0.366 |

***Note.*** For the higher severity group, the correlations with peer bonding could not be calculated, as most values (96%) were uniform (0). * indicates p<.05

In the lower severity NSSI group, by contrast, the association with Physicalism was invariably positive, and it was significant for three socially-relevant factors: Self-punishment (e.g., “when I self-harm, I am punishing myself”) Toughness (e.g., “seeing if I can stand the pain”) and Interpersonal influence (e.g., “letting others know the extent of my emotional pain”): The more Physicalist the participant was, the less likely they were to report these factors as relevant to their NSSI. None of these correlations, however, survived when adjusting for mindreading and autistic characteristics.

A.

B.

Altogether, then, these results suggest that, among people with more severe NSSI, those who veer towards Physicalism are less likely to attribute their self-harm to self-care, and these associations are not explained by differences in mind-reading and autistic characteristics. In the less severe subgroup, by contrast, once these covariates were controlled, there were no significant associations between ISAS functions and Physicalism.

2.3. An auxiliary analysis

The main text reports that, in the higher severity NSSI group, participants who veered toward Physicalism were less likely to endorse the Interpersonal factor as an explanation for their NSSI, and this association survived correction for autistic traits and mindreading ability. This correlation is of interest because it suggests that, within this sample, stronger Physicalism might be linked to the endorsement of the ISAS function.

As noted, however, these correlations only emerged once one outlier in the higher-severity group was removed. Given the small sample size of this subgroup (N=22), one might therefore worry that these conclusions could be unstable.

To evaluate this possibility, we examined the partial correlation after removing each of the remaining 21 participants, one participant at a time. Table S5 presents the results.

**Table S5.** Spearman’s partial correlations between Physicalism and the Interpersonal ISAS factor (Note: * indicates p<.05; N=21)

| **Participant removed** | | **Spearman's rho** | **p** | **Effect size (Fisher's z)** |
| --- | --- | --- | --- | --- |
| 1 | -0.4939* | | 0.037 | -0.541 |
| 2 | -0.5832* | | 0.011 | -0.667 |
| 4 | -0.5832* | | 0.011 | -0.667 |
| 8 | -0.5831* | | 0.011 | -0.667 |
| 11 | -0.5632* | | 0.015 | -0.638 |
| 12 | -0.465 | | 0.052 | -0.503 |
| 18 | -0.5614* | | 0.015 | -0.635 |
| 23 | -0.5673* | | 0.014 | -0.644 |
| 24 | -0.5967** | | 0.009 | -0.688 |
| 49 | -0.5400* | | 0.021 | -0.604 |
| 55 | -0.5186* | | 0.027 | -0.575 |
| 56 | -0.6031** | | 0.008 | -0.698 |
| 58 | -0.5286* | | 0.024 | -0.588 |
| 59 | -0.5291* | | 0.024 | -0.589 |
| 60 | -0.5574* | | 0.016 | -0.629 |
| 63 | -0.5059* | | 0.032 | -0.557 |
| 64 | -0.5933** | | 0.009 | -0.683 |
| 70 | -0.5745* | | 0.013 | -0.654 |
| 74 | -0.4962* | | 0.036 | -0.544 |
| 77 | -0.5273* | | 0.025 | -0.586 |
| 79 | -0.5634* | | 0.015 | -0.638 |

***Note.*** * p < .05, ** p < .01

An inspection of the results shows that 20 of these correlations remained significant, and for the remaining one (obtained after removing subject 12), *p=.052*. These results suggest that the unique, negative association between the tendency toward higher NSSI severity and the endorsement of the interpersonal factor is stable within this sample.

3. Auxiliary statistical analyses
   3.1. Modeling approach

Before exploring the statistical effect of NSSI on mind-body intuitions, we first sought to systematically evaluate which aspect of NSSI best captures the differences between the NSSI group and Controls. Informed by past research linking autistic characteristics to both intuitive Dualism*^4^*, and NSSI^6^, we further sought to determine whether the effects of NSSI remain after controlling for autistic characteristics (captured by the ASQ).

We approach this endeavor systematically, in two separate steps. The first step empirically identified which aspect of NSSI can best model our findings. In a second step, we compared multiple methods for modeling the effect of autistic characteristics and evaluated whether the “winner” further improves the fit of the NSSI model (relative to the “winner” of step 1).

*NSSI characteristics.* We first evaluated which characteristic of NSSI—its severity, recency, or group (NSSI vs. Control) alone—best fits the data. Severity was defined using a median split of the number of NSSI attempts over one’s lifetime: participants below the median were categorized as “Low”; those above the median were categorized as “High”. NSSI recency was captured by the presence of NSSI episodes in the month prior to testing (yes=1; no=0).

To determine which characteristic best improves the model’s fit, we first modeled the effect of Task (Afterlife vs. Replication) and Trait (Epistemic vs. Non-epistemic) alone within the glmer function of the lme4 package 4 (response ~ Task* Trait + (Trait|subject) + (1|item), family = “binomial”). In this model, task, and trait are fixed effects; subject and item are random effects. We refer to this basic version as V0.

Next, we compared whether group (NSSI vs. Control), Severity (High, Low, Control), or Recency (Recent, Non-recent, Control) yielded a better fit relative to V0 (when entered as fixed effects). The resulting models are as follows:

V0:	response ~ Task*Trait+ (Trait | subject) + (1 | item)

V1:	response ~ Task*Trait* **NSSI severity** **+** (Trait | subject) + (1 | item)

V2:	response ~ Task*Trait* **NSSI recency**+ (Trait | subject) + (1 | item)

V3	response ~ Task*Trait***Group**+ (Trait | subject) + (1 | item)

Models V1-V3 were each compared to V0 via an ANOVA. All four models (V0-V3) converged successfully (bobyqa optimizer, convergence code = 0) and showed no evidence of singularity (isSingular = FALSE); all models used a maximum of 800,000 function evaluations.

An inspection of the results (Table S6) further shows that only the severity model (V1) unambiguously improved the fit relative to V0, as it significantly improved X^2^ and reduced AIC, albeit these gains incurred some cost in BIC (i.e., a less optimal fit). The recency model (V2) yielded mixed results (better BIC, worse AIC), and it was not statistically significant. Accordingly, severity (V1) best captures group characteristics.

**Table S6**. ANOVA results, comparing the fit when adding NSSI characteristics into the basic regression model (V0)

| **Model** | **npar** | **AIC** | **BIC** | **logLik** | **Deviance** | **X^2^** | **Df** | **Pr(>Chisq)** |
| --- | --- | --- | --- | --- | --- | --- | --- | --- |
| V0 | 8 | 8459.4 | 8515.2 | -4221.7 | 8443.4 |  |  |  |
| V1 | 16 | 8448.2 | 8559.8 | -4208.1 | 8416.2 | 27.141 | 8 | 0.0006685 *** |
| V2 | 16 | 8460.8 | 8572.5 | -4214.4 | 8428.8 | 14.508 | 8 | 0.06944 |
| V3 | 12 | 8466.3 | 8550.0 | -4221.1 | 8442.3 | 1.098 | 4 | 0.8946 |

Note: Signif. codes: 0 ‘***’ 0.001 ‘**’ 0.01 ‘*’ 0.05 ‘.’ 0.1 ‘ ’ 1

a. The role of ASQ. Next, we examined whether the fit of the severity model (V1) improved by the addition of ASQ as a continuous factor, either as a covariate (in model V4) or as a moderator (in model V5). A third model, V6, entered ASQ as a categorical moderator; using a median split of the entire sample (NSSI and Controls), this categorical variable is dubbed “ASQ Level”. The three models are as follows:

V4: Response ~ Trait * Task * NSSI severity + ASQ + (Trait | subject) + (1 | item)

V5: Response ~ Trait * Task * NSSI severity * ASQ + (Trait | subject) + (1 | item)

V6: Response ~ Trait * Task * NSSI severity * ASQ_Level + (Trait | subject) + (1 | item)

Model V5 (with ASQ entered as a continuous moderator) failed to converge and was therefore excluded from model selection because its parameter estimates and fit statistics could not be considered reliable. In contrast, models V4 (with ASQ as a continuous covariate) and V6 (with ASQ as a categorical moderator) both converged successfully (bobyqa optimizer, convergence code = 0) and showed no evidence of singularity (isSingular = FALSE). We thus proceed to compare the remaining two candidates (V4 and V6) using likelihood ratio tests and information criteria (AIC and BIC).

Results (Table S7) favored the categorical moderator approach (V6) over the continuous moderator model (V4), as model V6 had a significantly better fit relative to the covariate continuous model (V4), and their complexity (AIC) was comparable.

We next compared model V6 with the original severity model (V1), which had no control for ASQ. Although the complexity of V6 was higher (evident in its larger BIC), AIC and the likelihood ratio test favored Model V6. Thus, the analysis in the main text reports the outcomes of model V6—with the categorical ASQ Level factor (lower vs. higher) entered as a moderator into the Severity model.

**Table S7.** ANOVA results comparing the categorical moderator modeling of ASQ (V6) to the continuous covariate model (V4) and the original severity model (without any control of ASQ, in V1).

| **Model** | **npar** | **AIC** | **BIC** |  | **logLik** | **Deviance** | **Chisq** | **Df** | **Pr(>Chisq)** |
| --- | --- | --- | --- | --- | --- | --- | --- | --- | --- |
| V4 | 17 | 8449.8 | 8568.4 |  | -4207.9 | 8415.8 |  |  |  |
| V6 | 28 | 8447.2 | 8642.6 |  | -4195.6 | 8391.2 | 24.56 | 11 | 0.01055 ** |
| V1 | 16 | 8448.2 | 8559.8 |  | -4208.1 | 8416.2 |  |  |  |
| V6 | 28 | 8447.2 | 8642.6 |  | -4195.6 | 8391.2 | 25.016 | 12 | 0.01475 * |

Note: Signifiance codes: 0 ‘***’ 0.001 ‘**’ 0.01 ‘*’ 0.05 ‘.’ 0.1 ‘ ’

3.2. Sensitivity analysis

To ensure that the link between NSSI severity and Physicalism (reported in the main text) was not due to the dichotomization of NSSI subgroups (into higher vs. lower, via median splitting), we conducted a sensitivity analysis in which NSSI status is defined continuously, via NSSI lifetime engagement (log-transformed; LN([X+1)). The model was response *(yes (1), no(0))* ~ Trait [epistemic, non-epistemic] * Task [body-replication, afterlife] * Lifetime NSSI + [Trait | subject] + [1 | item]).

**Table S8.** Logistic mixed-effects model with continuous NSSI coding.

| **Predictor** | ***β*** | ***SE*** | ***z*** | ***p*** |
| --- | --- | --- | --- | --- |
| Intercept | 0.622 | 0.199 | 3.13 | .002 |
| Task | 0.830 | 0.074 | 11.21 | < .001 |
| Trait | 0.016 | 0.211 | 0.08 | .939 |
| Lifetime NSSI | 0.018 | 0.077 | 0.23 | .818 |
| Task × Trait | 0.984 | 0.148 | 6.66 | < .001 |
| Task × Lifetime NSSI | 0.074 | 0.032 | 2.32 | .020 |
| Trait × Lifetime NSSI | −0.025 | 0.056 | −0.44 | .659 |
| Task × Trait × Lifetime NSSI | −0.034 | 0.063 | −0.53 | .593 |

**Note.** *β* = unstandardized regression coefficient; *SE* = standard error.

An inspection of the results (Table S8) confirms that, as in the omnibus model, Lifetime NSSI interacted with Task, such that greater NSSI severity was associated with stronger Physicalism (a more positive Task slope, defined as Replication - Afterlife). These results indicate that the association between NSSI and Physicalism is not confined to a particular definition of NSSI severity.

References

Appendix I: Experimental materials

a. Pre-screener
1. What is your age?
2. Where do you currently live?
3. Are you a native English speaker?
4. What is your sex at birth?
5. Do you have any reading or language disorder?
6. Have you received a formal clinical diagnosis of autism spectrum disorder, made by a psychiatrist, psychologist, or other qualified medical specialist? This includes Asperger’s syndrome, Autism Disorder, High Functioning Autism or Pervasive Developmental Disorder.
7. For the following questions, we are interested in drug and alcohol use. In the past month, how many times have you taken non-prescribed drugs OR had 5+ drinks of alcohol within one sitting?"
8. In the past year, how many times have you taken non-prescribed drugs OR

had 5+ drinks of alcohol within one sitting?

9. In your life, approximately how many times have you taken non-prescribed drugs OR had 5+ drinks of alcohol within one sitting?
10. Have you ever purposely hurt yourself without wanting to die?

For these questions, we are referring to times where you have directly hurt yourself without wanting to die, for example, like cutting or burning your skin. We are not asking about times where you have indirectly hurt yourself, for example times where you starved yourself, overdosed without wanting to die, got a tattoo/piercing.

11. Approximately how many separate times in the past month have you hurt yourself without wanting to die? Please only include those times that drew blood or left a mark lasting for at least a few days.
12. Approximately how many separate times in the past 5 years have you hurt yourself without wanting to die? Please enter an exact value below. Please only include those times that drew blood or left a mark lasting for at least a few days.
13. Approximately how many separate times in your life* have you purposely hurt yourself without wanting to die? Please enter an exact value below. Please only include those times that drew blood or left a mark lasting for at least a few days.

*For some people the number of lifetime episodes can be difficult to estimate. If this number is hard for you to guess, grab a piece of paper and a pen and try to take the following steps:

a. Think back to the year when you were most frequently hurting yourself without wanting to die. How many times did you do each of the things that year? (Write the number down)
b. Try to think of the average number of times per year you have engaged in each of these behaviors. (Write this number down)
c. Calculate the number of years you've engaged in these behaviors minus 1.
d. Multiply the ""average"" from step 2 by the number you calculated in step 3. (Write the number down)
e. Add that number to the first number you estimated (that is, the number of episodes from the year that you were most frequently hurting yourself without wanting to die that year) to obtain the lifetime episode estimate."
b. List of psychological traits used in the Dualism task

|  | **Category** | **Trait** |
| --- | --- | --- |
| 1. | Non-epistemic | Anger in response to hostility |
| 2. | Non-epistemic | Love for one’s family |
| 3. | Non-epistemic | Contentment with one’s life |
| 4. | Non-epistemic | Disgust by feces |
| 5. | Non-epistemic | Excitement towards an opportunity |
| 6. | Non-epistemic | Fear of danger |
| 7. | Non-epistemic | Happiness at the birth of one’s child |
| 8. | Non-epistemic | Joy of being |
| 9. | Non-epistemic | Pride in one’s accomplishments |
| 10. | Non-epistemic | Sadness from a friend’s death |
| 11. | Non-epistemic | Shame from one’s shortcomings |
| 12. | Non-epistemic | Surprise at an unexpected event |
| 13. | Non-epistemic | Trust in one’s family |
| 14. | Non-epistemic | Jealousy towards a lover |
| 15. | Non-epistemic | Envy at a competitor’s success |
| 16. | Non-epistemic | Empathy towards a person in need |
| 17. | Non-epistemic | Admiration for wisdom |
| 18. | Non-epistemic | Pain from witnessing illness and death |
| 19. | Non-epistemic | Hope for a better future |
| 20. | Non-epistemic | Affection towards others |
| 21. | Non-epistemic | Gripping objects by hand |
| 22. | Non-epistemic | Sitting down to relax |
| 23. | Non-epistemic | Walking to move around |
| 24. | Non-epistemic | Running when in a hurry |
| 25. | Non-epistemic | Kicking with one’s feet |
| 26. | Non-epistemic | Lifting objects with hands |
| 27. | Non-epistemic | Stretching one’s muscles |
| 28. | Non-epistemic | Licking with one’s tongue |
| 29. | Non-epistemic | Yawning when tired |
| 30. | Non-epistemic | Breathing heavily after exertion |
| 31. | Non-epistemic | Squatting down |
| 32. | Non-epistemic | Trembling at cold temperatures |
| 33. | Non-epistemic | Tickling a child to make them laugh |
| 34. | Non-epistemic | Touching other people with one’s hands |
| 35. | Non-epistemic | Smelling the scent of food |
| 36. | Non-epistemic | Sleeping to restore one’s energy |
| 37. | Non-epistemic | Seeing objects with one’s eyes |
| 38. | Non-epistemic | Sniffling when one has a cold |
| 39. | Non-epistemic | Crying at sad events |
| 40. | Non-epistemic | Dancing at to a rhythm |
| 41. | Epistemic | Recalling past events |
| 42. | Epistemic | Judging one’s options |
| 43. | Epistemic | Distinguishing between right and wrong |
| 44. | Epistemic | Reflecting on one’s past and future |
| 45. | Epistemic | Having self control of one’s own behavior |
| 46. | Epistemic | Speculating about the possible outcomes of events |
| 47. | Epistemic | Making jokes |
| 48. | Epistemic | Thinking about magic |
| 49. | Epistemic | Using metaphors |
| 50. | Epistemic | Mourning the dead |
| 51. | Epistemic | Observing rituals |
| 52. | Epistemic | Overcoming a fear |
| 53. | Epistemic | Recognizing taboos |
| 54. | Epistemic | Recognizing relations among kin |
| 55. | Epistemic | Interpreting others' behaviors |
| 56. | Epistemic | Symbolic reasoning |
| 57. | Epistemic | Making comparisons |
| 58. | Epistemic | Keeping track of time |
| 59. | Epistemic | Planning for the future |
| 60. | Epistemic | Recognizing melodies |
| 61. | Epistemic | Keeping track of people’s age |
| 62. | Epistemic | Forming sentences |
| 63. | Epistemic | Forming words |
| 64. | Epistemic | Abstract reasoning |
| 65. | Epistemic | Having preferences concerning aesthetics |
| 66. | Epistemic | Attributing human qualities to inanimate objects |
| 67. | Epistemic | Having a belief in the supernatural |
| 68. | Epistemic | Having beliefs about fortune and misfortune |
| 69. | Epistemic | Devising classification of body parts |
| 70. | Epistemic | Having classification of animals |
| 71. | Epistemic | Having classification of plants |
| 72. | Epistemic | Having classification of weather |
| 73. | Epistemic | Devising methods to heal the sick |
| 74. | Epistemic | Having a contrast between 'general' and 'particular' |
| 75. | Epistemic | Having a logical notion of 'and' |
| 76. | Epistemic | Having a logical notion of 'not' |
| 77. | Epistemic | Forming myths |
| 78. | Epistemic | Having a concept of 'person' |
| 79. | Epistemic | Having a preference for one's own children and close kin |
| 80. | Epistemic | Having norms about trade |

c. **Instructions in the body-replication task**

In what follows, we are asking you to reason about a hypothetical scenario.

Suppose it were possible to grow a replica of the body of an adult human donor. The replica preserves every aspect of the human body and brain. In particular, suppose that the body of the replica looks and works precisely like a normal human body. Similarly, the brain replica is identical in all respects to the brain of the adult donor.

Below is a list of various traits that define the human donor. Will these traits emerge in its replica?

Please indicate your answer as either 1=yes, this trait will emerge in the replica OR 2=no, this trait will not emerge in the replica. Thank you!

[A photo suggestive of body-replication is shown for illustrative purposes. The photo includes two panels. The left panel features a young man; the right panel features an anatomical diagram of a similar young man. The two panels are linked by an arrow, suggestive of a reduplication procedure.]

d. **Instructions in the afterlife task**

In this experiment, we examine people’s beliefs about the afterlife. Whether or not there is an afterlife, is of course unknown. But for the purpose of this experiment, we invite you to assume that after people die, they do continue to exist in some capacity. Your task is to reason about which human traits are likely to be maintained in the afterlife.

Below is a list of human traits. Then, please determine the following: if an afterlife exists, will these traits emerge in the afterlife?

Please indicate your answer as either 1=yes, this trait will emerge in the afterlife OR 2=no, this trait will not emerge in the afterlife.  Thank you!

[A photo suggestive of the afterlife is shown for illustrative purposes. The photo features a person in a tunnel, walking towards bright light. ]

e. **Instructions for the ToM task (Adapted from Dodell-Feder et al., 2011)**

We typically go about our everyday life guided by our beliefs about how things are, and about what other people think. Sometimes these beliefs are true; but at times, they are not.

In this experiment, you will be presented with short stories, describing a person and their beliefs. We ask you to indicate whether this belief is true or false. Please respond as quickly and accurately as you can.

Thank you!

f. **Materials in the ToM task (Adapted from Dodell-Feder et al.,** 2011)

|  | Story | Answer |
| --- | --- | --- |
| 1. | The morning of high school dance Sarah placed her high heel shoes under her dress and then went shopping. That afternoon, her sister borrowed the shoes and later put them under Sarah's bed. *Sarah gets ready assuming her shoes are under the dress.* | Yes |
| 2. | John told Mary that he had lost his keys, and the two of them go searching. While John looks outside, Mary finds the keys, but since she’s in a hurry, she just places them on the table at the entrance and leaves. *When John returns, he expects to see the keys on the table.* | No |
| 3. | Expecting the game to be suspended because of the rain, the Garcia family took the subway home. The score was tied, 3-3, and on their way home, the family had no internet connection. During their commute the rain stopped and the game soon ended with a score of 5-3. *The Garcia family arrives home believing the score is 5-3.* | No |
| 4. | Susie parked her sports car in the driveway. In the middle of the night, Nathan moved her car into the garage to make room for his minivan. When Susie wakes up, *she expects to see her car in the driveway.* | Yes |
| 5. | When Lisa left Jacob, he was deep asleep on the beach. A few minutes later a wave woke him. Seeing Lisa was gone, Jacob decided to go swimming. *Lisa now believes that Jacob is sleeping.* | Yes |
| 6. | Larry chose a debated topic for his class paper due on Friday. The news on Thursday indicated that the debate had been solved, but Larry never read it. *When Larry writes his paper he thinks the debate has been solved.* | No |
| 7. | A window wiper was commissioned by a CEO to wipe an entire building. He finished the right side, but his platform gets stuck before he could do the left side, so he reports the problem to the CEO. Overnight, the cleaner comes up with a solution, and early in the morning, he diligently returns to finish the job. *When the CEO comes to work at noon, he expects to see all of the windows are cleaned.* | No |
| 8. | Sally and Greg called ahead of time to make a reservation for the back-country cabin. The park ranger forgot to write down the reservation and two other hikers got to the cabin first. *When the hikers arrive, they expect the other hikers to be in their room.* | No |
| 9. | Rather than driving to work, today Amy decided to walk. When George woke up, he saw her car in the drive. Amy’s room was quiet and dark. George knows that when Amy is sick, she lies down in a dark room. *George expects Amy is in her room, sick with migraine.* | Yes |
| 10. | Laura didn't have time to braid her horse’s mane before going to camp. While she was at camp, William brushed Laura's horse and braided the horse’s mane for her. *Laura returns assuming that her horse's hair isn't braided.* | Yes |
| 11. | At night a bear broke into a cooler near a tent and drank the soda. Five hours later, the campers woke up and went to their cooler for breakfast. *In the cooler, the campers expect to find soda.* | Yes |
| 12. | Anne made lasagna in the blue dish. After Anne left, Ian came home and ate the lasagna. Then he filled the blue dish with spaghetti and replaced it in the fridge. *Anne thinks the blue dish contains spaghetti.* | No |
| 13. | Jenny put her chocolate away in the cupboard. Then she went outside. Alan moved the chocolate from the cupboard into the fridge. Half an hour later, Jenny came back inside. *Jenny expects to find her chocolate in the cupboard.* | Yes |
| 14. | The weather was so warm today that all the tulips in Pam's backyard suddenly bloomed. The tulips next to Pam's office still have not yet flowered, though. Pam has been at work all day. *Driving home after work, Pam supposes her tulips have not bloomed.* | Yes |
| 15. | When the class' science test was handed back, Shannon was mistakenly given Adam's test. A large B was written on the front of Adam's test, but Shannon's actual grade was an A. *Shannon believes she received a B on the exam.* | Yes |
| 16. | Every day Jill goes to the coffee shop on the corner and orders a latte, her favorite drink. Today, the cashier misunderstands Jill and prepares a mocha instead. *Jill thinks her drink will taste like a mocha.* | No |
| 17. | Hopeful to catch a prize fish, George went fishing. That afternoon, he saw his fishing line bend over as if he had caught a big fish. Actually, George’s fishing pole had snagged a small tire. *At the end of the fishing line, George expects to see a tire.* | No |
| 18. | The girls left ice cream in the freezer before they went to sleep. Overnight, the power to the kitchen was cut and the ice cream melted. *When they get up the girls believe the ice cream is melted.* | No |
| 19. | Ken told Andrea that he was going shopping for sandals. At the shoe store, Ken noticed a very nice pair of boots on sale, and bought them instead. *When he meets Andrea, she believes Ken's shoe store bag contains boots*. | No |
| 20. | Jeff is colorblind, so he cannot tell subtle differences in color. To help him dress up, his housekeeper normally keeps the pink and white shirts in different drawers, but this week, she is sick, and her replacement placed the white shirts at the bottom drawer, where the pink shirts usually are. *When Jeff reaches out to the bottom drawer and puts on a shirt, he believes the shirt is pink.* | Yes |

g. **Instructions and materials in the ASQ (from ^1^ )**

For each statement below, choose one response that best describes how strongly that statement applies to you. Responses are given as: Definitely Agree; Slightly Agree; Slightly Disagree; Definitely Disagree

1. I prefer to do things with others rather than on my own.

2. I prefer to do things the same way over and over again.

3. If I try to imagine something, I find it very easy to create a picture in my mind.	4. I frequently get so strongly absorbed in one thing that I lose sight of other things.	5. I often notice small sounds when others do not.

6. I usually notice car number plates or similar strings of information.

7. Other people frequently tell me that what I’ve said is impolite, even though I think it is polite.

8. When I’m reading a story, I can easily imagine what the characters might look like.	9. I am fascinated by dates.

10. In a social group, I can easily keep track of several different people’s

conversations.

11. I find social situations easy.

12. I tend to notice details that others do not.

13. I would rather go to a library than to a party.

14. I find making up stories easy.

15. I find myself drawn more strongly to people than to things.

16. I tend to have very strong interests, which I get upset about if I can’t pursue.	17. I enjoy social chitchat.

18. When I talk, it isn’t always easy for others to get a word in edgewise.

19. I am fascinated by numbers.

20. When I’m reading a story, I find it difficult to work out the characters’ intentions.	21. I don’t particularly enjoy reading fiction.

22. I find it hard to make new friends.

23. I notice patterns in things all the time.

24. I would rather go to the theater than to a museum.

25. It does not upset me if my daily routine is disturbed.

26. I frequently find that I don’t know how to keep a conversation going.

27. I find it easy to “read between the lines” when someone is talking to me.	28. I usually concentrate more on the whole picture, rather than on the small details.	29. I am not very good at remembering phone numbers.

30. I don’t usually notice small changes in a situation or a person’s appearance.	31. I know how to tell if someone listening to me is getting bored.	32. I find it easy to do more than one thing at once.

33. When I talk on the phone, I’m not sure when it’s my turn to speak.	34. I enjoy doing things spontaneously.

35. I am often the last to understand the point of a joke.

36. I find it easy to work out what someone is thinking or feeling just by looking at

their face.

37. If there is an interruption, I can switch back to what I was doing very quickly.	38. I am good at social chitchat.

39. People often tell me that I keep going on and on about the same thing.

40. When I was young, I used to enjoy playing games involving pretending with

other children.

41. I like to collect information about categories of things (e.g., types of cars, birds,

trains, plants).

42. I find it difficult to imagine what it would be like to be someone else.

43. I like to carefully plan any activities I participate in.

44. I enjoy social occasions.

45. I find it difficult to work out people’s intentions.

46. New situations make me anxious.

47. I enjoy meeting new people.

48. I am a good diplomat.

49. I am not very good at remembering people’s date of birth.

50. I find it very easy to play games with children that involve pretending.

h. **Instructions and materials in the ISAS (Klonsky & Glenn, 2009)**

**SECTION I. BEHAVIOR**

**Instructions**

This questionnaire asks about a variety of self-harm behaviors. Please only endorse a behavior if you have done it intentionally (i.e., on purpose) and without suicidal intent (i.e., not for suicidal reasons).

1. Please estimate the number of times in your life you have intentionally (i.e., on purpose) performed each type of non-suicidal self-harm (e.g., 0, 10, 100, 500):

| Cutting ___ | Severe Scratching ___ |
| --- | --- |
| Biting ___ | Banging or Hitting Self ___ |
| Burning ___ | Interfering w/ Wound Healing (e.g., picking scabs) ___ |
| Carving ___ | Rubbing Skin Against Rough Surface ___ |
| Pinching ___ | Sticking Self with Needles ___ |
| Pulling Hair ___ | Swallowing Dangerous Substances ___ |
| Other ___, ___ |  |

2. If you feel that you have a *main* form of self-harm, please circle the behavior(s) on the first page above that you consider to be your main form of self-harm.
3. At what age did you:

- First harm yourself? ____
- Most recently harm yourself? (approximate date – month/date/year) ____

4. Do you experience physical pain during self-harm?
   a. YES
   b. SOMETIMES
   c. NO
5. When you self-harm, are you alone?
   a. YES
   b. SOMETIMES
   c. NO
6. Typically, how much time elapses from the time you have the urge to self-harm until you act on the urge?
   a. < 1 hour
   b. 1 - 3 hours
   c. 3 - 6 hours
   d. 6-12 hours
   e. 12-24 hours
   f. > 1 day
7. Do you want to stop self-harming?
   a. YES
   b. NO

**SECTION II. FUNCTIONS**

**Instructions**

This inventory was written to help us better understand the experience of non-suicidal self-harm. Below is a list of statements that may or may not be relevant to your experience of self-harm. Please identify the statements that are most relevant for you:

- Circle **0** if the statement is **not relevant** for you at all
- Circle **1** if the statement is **somewhat relevant** for you
- Circle **2** if the statement is **very relevant** for you

“When I self-harm, I am …

1. …calming myself down
2. …creating a boundary between myself and others
3. …punishing myself
4. …giving myself a way to care for myself (by attending to the wound)
5. …causing pain so I will stop feeling numb
6. …avoiding the impulse to attempt suicide
7. …doing something to generate excitement or exhilaration
8. …bonding with peers
9. …letting others know the extent of my emotional pain
10. …seeing if I can stand the pain
11. …creating a physical sign that I feel awful
12. …getting back at someone
13. …ensuring that I am self-sufficient
14. …releasing emotional pressure that has built up inside of me
15. …demonstrating that I am separate from other people
16. …expressing anger towards myself for being worthless or stupid
17. …creating a physical injury that is easier to care for than my emotional

distress

18. …trying to feel something (as opposed to nothing) even if it is physical

pain

19. …responding to suicidal thoughts without actually attempting suicide
20. …entertaining myself or others by doing something extreme
21. …fitting in with others
22. …seeking care or help from others
23. …demonstrating I am tough or strong
24. …proving to myself that my emotional pain is real
25. …getting revenge against others
26. …demonstrating that I do not need to rely on others for help
27. …reducing anxiety, frustration, anger, or other overwhelming emotions
28. …establishing a barrier between myself and others
29. …reacting to feeling unhappy with myself or disgusted with myself
30. …allowing myself to focus on treating the injury, which can be

gratifying or satisfying

31. …making sure I am still alive when I don’t feel real
32. …putting a stop to suicidal thoughts
33. …pushing my limits in a manner akin to skydiving or other extreme

activities

34. …creating a sign of friendship or kinship with friends or loved ones
35. …keeping a loved one from leaving or abandoning me
36. …proving I can take the physical pain
37. …signifying the emotional distress I’m experiencing
38. …trying to hurt someone close to me
39. …establishing that I am autonomous/independent
i. **Demographics survey**
1. **Please select your age range**.
   a. Under 18
   b. 18-25
   c. 25-30
   d. 30+
2. **Please specify your exact age**. ______
3. **What is your sex (assigned at birth)?**
   a. Female
   b. Male
   c. Intersex
   d. Prefer Not to Say
4. **What is your gender identity?**
   a. Female
   b. Male
   c. Transgender Female
   d. Transgender Male
   e. Non-binary/genderqueer
   f. Prefer Not to Say
   g. Other (please specify): ______
5. **What is your race? Select all that apply.**
   a. White
   b. Black or African American
   c. Native American or Alaska Native
   d. Asian
   e. Native Hawaiian or Pacific Islander
   f. Prefer Not to Say
   g. Not listed here (please specify): ______
6. **What is your sexual orientation?**
   a. Asexual
   b. Bisexual
   c. Heterosexual/Straight
   d. Homosexual (Gay or Lesbian)
   e. Pansexual
   f. Unsure
   g. Prefer Not to Say
   h. Not listed here (please specify): ______
7. **What is your employment status?**
   a. Employed full time (40 or more hours per week)
   b. Employed part time (up to 39 hours per week)
   c. Unemployed and currently looking for work
   d. Unemployed and not currently looking for work
   e. Student
   f. Retired
   g. Stay-at-home
   h. Unable to work
   i. On parental leave
8. **What is the highest level of education you have received?**
   a. High School / GED
   b. Associates Degree
   c. Bachelors Degree
   d. Masters Degree
   e. Doctorate Degree
   f. Other (please specify): ______

**Appendix II: Annotated R Code**

\# Load packages.

library(lme4)

library(tidyr)

library(dplyr)

library(emmeans)

\# Note: This R script uses relative paths and assumes that the script and the data file ("Dualism_shared.txt") are saved in the same directory.

\# To run this script:

\# Option 1: In RStudio, open this script and select:

\# Session --> Set Working Directory --> To Source File location.

\# Option 2: Uncomment the line below and specify the working directory manually (revising the path below to the folder containing both the R script and data file:

\# setwd("path/to/your/folder")

\# Verify current working directory:

getwd()

\# Then import the data:

 dat = read.table("Dualism_shared.txt",stringsAsFactors = FALSE, fill=TRUE, sep = "\t", header= T)

\# Code vector type.

dat$subject <- as.factor(dat$subject)

dat$trait <- as.factor(dat$trait)

dat$group <- as.factor(dat$group)

dat$acc <- as.numeric(dat$acc)

dat$item <- as.factor(dat$item)

dat$Task <- as.factor(dat$Task)

dat$recency <- as.factor(dat$recency)

dat$severity <- as.factor(dat$severity)

dat$ASQ_Level <- as.factor(dat$ASQ_Level)

dat$Sex <- as.factor(dat$Sex)

\# Count subjects and items by condition.

n_distinct(dat$subject) # This shows 99 unique subjects.

n_distinct(dat$item)

dat %>% group_by(group, Task,trait,severity) %>% summarise(count = n_distinct(item))

n_distinct(dat$subject)

dat %>% group_by(group, Task,trait,severity) %>% summarise(count = n_distinct(subject))

#make sure there's no duplicates

(dat %>% group_by(trait, subject, item) %>% summarize(n=length(subject)) %>% filter(n>1))

\# Prepare data file for analysis (Note: this line is a relic from an older code; it does not actually remove the Control group, but it is still necessary for this code to work/

dat.alldata = dat[dat$trait!="Control",]

dat.contrasts <- dat.alldata

\# This confirms original treatment coding.

contrasts(dat.contrasts$trait)

contrasts(dat.contrasts$group)

contrasts(dat.contrasts$Task)

contrasts(dat.contrasts$recency)

contrasts(dat.contrasts$severity)

contrasts(dat.contrasts$ASQ_Level)

contrasts(dat.contrasts$Sex)

\# set and confirm coding

contrasts(dat.alldata$group) <- cbind(c(-.5, .5))

contrasts(dat.alldata$trait) <- cbind(c(-.5, .5))

contrasts(dat.alldata$Task) <- cbind(c(-.5, .5))

contrasts(dat.alldata$ASQ_Level) <- cbind(c(-.5, .5))

contrasts(dat.alldata$Sex) <- cbind(c(-.5, .5))

contrasts(dat.alldata$group)

contrasts(dat.alldata$trait)

contrasts(dat.alldata$Task)

contrasts(dat.alldata$recency)

contrasts(dat.alldata$severity)

contrasts(dat.alldata$ASQ_Level)

contrasts(dat.alldata$Sex)

#FINAL MODEL REPORTED IN SECTION 3.1 OF THE MAIN TEXT

dat.alldata.glmm.v6 <- glmer(acc ~ trait * Task * severity * ASQ_Level + (trait | subject) + (1 |	item),

data = dat.alldata,

family = binomial,

control = glmerControl(optimizer="bobyqa", optCtrl = list(maxfun = 800000)))

summary(dat.alldata.glmm.v6)

emm <- emmeans(dat.alldata.glmm.v6, ~ Task | severity * ASQ_Level)

contrast(emm, method = "revpairwise")

\# THIS SECTION DETAILS THE STEPS TAKEN TO IDENTIFY THE MODEL ABOVE (V6) AS THE BEST FITTING MODEL

#STEP 1: RUN DIFFERENT MODELS OF NSSI (SEVERITY, RECENCY) AND COMPARE THEM TO V0 (WITHOUT ANY PARTICIPANT CHARACTERISTICS)

\# New (trait | item): NO PARTICIPANT CHARCTERISTICS

dat.alldata.glmm.v0 <- glmer(acc ~ Task*trait+ (trait | subject) + (1 | item),

data = dat.alldata,

family = binomial,

control = glmerControl(optimizer="bobyqa", optCtrl = list(maxfun = 800000)))

summary(dat.alldata.glmm.v0)

dat.alldata.glmm.v0@optinfo$conv

cat("Singular fit:", isSingular(dat.alldata.glmm.v0), "\n")

#NSSI SEVERITY

dat.alldata.glmm.v1 <- glmer(acc ~ Task*trait*severity+ (trait | subject) + (1 | item),

data = dat.alldata,

family = binomial,

control = glmerControl(optimizer="bobyqa", optCtrl = list(maxfun = 800000)))

summary(dat.alldata.glmm.v1)

dat.alldata.glmm.v1@optinfo$conv

cat("Singular fit:", isSingular(dat.alldata.glmm.v1), "\n")

#NSSI RECENCY

dat.alldata.glmm.v2 <- glmer(acc ~ Task*trait*recency+ (trait | subject) + (1 | item),

data = dat.alldata,

family = binomial,

control = glmerControl(optimizer="bobyqa", optCtrl = list(maxfun = 800000)))

summary(dat.alldata.glmm.v2)

dat.alldata.glmm.v2@optinfo$conv

cat("Singular fit:", isSingular(dat.alldata.glmm.v2), "\n")

#GROUP (CONTRAST NSSI VS. CONTROL)

dat.alldata.glmm.v3 <- glmer(acc ~ Task*trait*group+ (trait | subject) + (1 | item),

data = dat.alldata,

family = binomial,

control = glmerControl(optimizer="bobyqa", optCtrl = list(maxfun = 800000)))

summary(dat.alldata.glmm.v3)

dat.alldata.glmm.v3@optinfo$conv

cat("Singular fit:", isSingular(dat.alldata.glmm.v3), "\n")

#STEP 2: CONTRAST THE DIFFERENT NSSI MODELS WITH V0

anova(dat.alldata.glmm.v0, dat.alldata.glmm.v1)

anova(dat.alldata.glmm.v0, dat.alldata.glmm.v2)

anova(dat.alldata.glmm.v0, dat.alldata.glmm.v3)

dat.alldata.glmm.v1 <- glmer(acc ~ Task*trait*severity+ (trait | subject) + (1 | item),

data = dat.alldata,

family = binomial,

control = glmerControl(optimizer="bobyqa", optCtrl = list(maxfun = 800000)))

summary(dat.alldata.glmm.v1)

dat.alldata.glmm.v1.1 <- glmer(acc ~ Task*trait*severity+ASQ+ (trait | subject) + (1 | item),

data = dat.alldata,

family = binomial,

control = glmerControl(optimizer="bobyqa", optCtrl = list(maxfun = 800000)))

summary(dat.alldata.glmm.v1.1)

dat.alldata.glmm.v1.2 <- glmer(acc ~ Task*trait*severity*ASQ+ (trait | subject) + (1 | item),

data = dat.alldata,

family = binomial,

control = glmerControl(optimizer="bobyqa", optCtrl = list(maxfun = 800000)))

summary(dat.alldata.glmm.v1.2)

anova(dat.alldata.glmm.v1, dat.alldata.glmm.v1.1)

anova(dat.alldata.glmm.v1, dat.alldata.glmm.v1.2)

#STEP 3: Add autistic characteristics, coded either continuously or categorically, as covariates or moderators

#v4: AQ coded as a continuous variable, entered as a covariate

dat.alldata.glmm.v4 <- glmer(acc ~ trait * Task*severity+ASQ+ (trait | subject) + (1 | item),

data = dat.alldata,

family = binomial,

control = glmerControl(optimizer="bobyqa", optCtrl = list(maxfun = 800000)))

summary(dat.alldata.glmm.v4)

dat.alldata.glmm.v4@optinfo$conv

cat("Singular fit:", isSingular(dat.alldata.glmm.v4), "\n")

#v5: AQ coded continuously, entered as a moderator

dat.alldata.glmm.v5 <- glmer(acc ~ trait * Task*severity*ASQ+ (trait | subject) + (1 | item),

data = dat.alldata,

family = binomial,

control = glmerControl(optimizer="bobyqa", optCtrl = list(maxfun = 800000)))

summary(dat.alldata.glmm.v5)

dat.alldata.glmm.v5@optinfo$conv

cat("Singular fit:", isSingular(dat.alldata.glmm.v5), "\n")

#V6: AQ coded categorically, and entered as a moderator

dat.alldata.glmm.v6 <- glmer(acc ~ trait * Task*severity*ASQ_Level+ (trait | subject) + (1 | item),

data = dat.alldata,

family = binomial,

control = glmerControl(optimizer="bobyqa", optCtrl = list(maxfun = 800000)))

summary(dat.alldata.glmm.v6)

#STEP 4: DETERMINE THE OPTIMAL MODEL OF AQ, BY COMPARING THE TWO CONVERGING AQ MODELS (V4, V6) TO EACH OTHER, AND TO V1 (SEVERITY, WITHOUT AQ)

anova(dat.alldata.glmm.v4, dat.alldata.glmm.v6)

anova(dat.alldata.glmm.v4, dat.alldata.glmm.v1)

anova(dat.alldata.glmm.v6, dat.alldata.glmm.v1)

#coef(dat.alldata.glmm)
